# Understanding vulnerability to flood-induced disasters: a comprehensive scoping review on at-risk individuals and evacuation challenges

**DOI:** 10.1186/s12913-025-13898-w

**Published:** 2025-12-18

**Authors:** Clara Del Prete, Martina Valente, Abesha Mitiku Saji, Amir Khorram-Manesh, Luca Ragazzoni

**Affiliations:** 1 CRIMEDIM, Center for Research and Training in Disaster Medicine, Humanitarian Aid and Global Health, Università del Piemonte Orientale, Via Lanino 1, 28100 Novara, Italy; 2 Department for Sustainable Development and Ecological Transition, Università del Piemonte Orientale, 13100 Vercelli, Italy; 3 Department of Health Sciences and Translational Medicine, Università del Piemonte Orientale, 13100 Vercelli, Italy; 4 Department of Surgery, Institute for Clinical Sciences, Sahlgrenska Academy, Gothenburg University, Gothenburg, Sweden; 5 Disaster Medicine Centre, Sahlgrenska Academy, Gothenburg University, Gothenburg, Sweden

**Keywords:** Vulnerability, Floods, Disaster management, Evacuation, Sheltering

## Abstract

Flood-induced disasters are increasing in frequency and severity, with high-income countries (HICs) facing growing health, social, and operational challenges, particularly in managing and protecting vulnerable populations that are disproportionately affected. This scoping review, conducted in accordance with PRISMA-ScR guidelines, examined how vulnerability is defined, addressed, and operationalized in evacuation management during flood-induced disasters in HICs. A systematic search identified 98 peer-reviewed studies published between 2014 and 2025. The analysis revealed critical gaps and inconsistencies in how vulnerability is conceptualized and applied in evacuation contexts. Several groups–including single-parent households, caregivers, individuals with mental health conditions, undocumented workers, and those facing linguistic or educational barriers–remain underrepresented, while others, such as informal caregivers, religious minorities, and victims of domestic abuse, are not addressed at all. The review also identified substantial variation in national approaches to vulnerability mapping and management, with most countries relying on fragmented and inconsistently integrated practices. Evidence linking vulnerability assessment to evacuation planning and sheltering decisions remains limited. Research on Flexible Surge Capacity and the use of Alternative Care Facilities for vulnerability-informed evacuation remains scarce, particularly regarding non-medical facilities as community-based hubs for rapid assessment and triage. Furthermore, few studies addressed continuity across the disaster timeline, linking pre-disaster vulnerability assessment, rapid needs assessment, and long-term recovery support. This review advances understanding of how vulnerability can be more effectively embedded in evacuation management, providing empirically grounded and comparative insights to inform inclusive, intersectional, and operational flood-induced disaster management strategies in HICs.

**Supplementary Information:**

The online version contains supplementary material available at 10.1186/s12913-025-13898-w.

## Background

Flood-induced disasters–caused by flash and coastal floods, storm surges, and extreme precipitation [1, 2]–are increasing in frequency and intensity due to climate change. Exposure and vulnerability to these hazards are also rising due to rapid uncontrolled urban development and population growth in flood-prone areas [3–5], thus leading to an overall surge in the risk of flood-induced disasters. Mounting evidence from recent events has highlighted the urgent need for strengthened flood management and health system resilience [6, 7]. Although mortality related to flood-induced disasters has decreased in High-Income Countries (HICs) [4, 8, 9], these events remain the most frequent worldwide, causing widespread health, social, and economic disruptions [4, 10]. Flood-induced disasters compromise health and social care systems, hindering service continuity and delaying or preventing access to care [11, 12]. These disruptions often trigger secondary surges of health needs: delayed but substantial increases in healthcare demand due to unmet needs and exacerbated conditions [13]. The overall health burden of flood-induced disasters is typically much greater in Low- and Middle-Income Countries, where limited infrastructure and constrained capacity to prepare, respond and recover from these events hinder effective disaster management [4, 8, 9]. In HICs, reported health effects include respiratory and dermatological infections, waterborne and faecal-oral diseases, injuries, mental health issues, and the worsening of chronic conditions [11, 14–16]. However, the impacts are not evenly distributed: individuals facing intersecting disadvantages are disproportionately affected [14, 15, 17]. The European Union (EU) Preparedness Strategy underscores the need for integrated and inclusive disaster management strategies tailored to vulnerable populations’ needs [18]. The United Nations Office for Disaster Risk Reduction defines vulnerability as “the conditions determined by physical, social, economic and environmental factors or processes which increase the susceptibility of an individual, a community, assets or systems to the impacts of hazards” [19]. While this multidimensional nature of vulnerability has also been acknowledged in previous studies [20–23], much of the existing literature remains fragmented and either overly specific, overly broad or lacking operational relevance for real-world disaster management applications [14, 21, 24–33]. Traditional approaches have conceptualized vulnerability as static, failing to account for contextual and situational factors. In contrast, recent literature emphasizes its dynamic, multidimensional and intersectional nature [34]. Vulnerability is increasingly understood not as a fixed lable but as a layered condition shaped by evolving individual, social and structural factors [35]. Recent contributions further highlight the importance of integrating both quantitative and qualitative methods to better capture context-specific and locally relevant vulnerabilities [36, 37]. However, although this reconceptualization is gaining traction theoretically, many empirical studies continue to rely on predefined group-based categories due to data constraints and methodological limitations, thus limiting the integration of more nuanced perspectives into the operational domain of disaster risk management. This disconnect underscores the need for practice-oriented frameworks capable of translating theoretical complexity into actionable planning and decision-making processes.

Several recent scoping reviews have sought to consolidate approaches to vulnerability assessment across hazards and disciplines. A systematic synthesis of social vulnerability indices revealed extensive reliance on pre-existing models, limited methodological innovation, and weak contextual adaptation [36]. Another large-scale review mapped nearly 300 indices across health, environmental, and disaster research, showing convergence around common domains such as age, education, and socioeconomic status, despite disciplinary differences [38]. A further methodological assessment identified significant inconsistencies in indicator weighting and scale sensitivity, highlighting the need for more robust and theoretically grounded approaches to vulnerability measurement [39]. Another systematic scoping review identified 21 core domains and called for dynamic, land-integrated, and methodologically diverse indicators to enhance the applicability of vulnerability indices [40]. Complementing these quantitative assessments, a One Health–oriented review underscored the value of participatory and context-sensitive methods to integrate social and ecological dimensions of vulnerability [37]. While these reviews provide valuable methodological insights, their primary focus remains on index development and vulnerability measurement rather than the application of vulnerability concepts to specific phases of disaster management.

In addition to these conceptual and methodological gaps, a critical shortcoming concerns the limited attention to how vulnerability is addressed during evacuation–a high-risk, operationally demanding phase of the disaster management. There is currently no comprehensive synthesis examining how vulnerability is defined, identified, or accommodated during flood-induced disaster evacuations, particularly in high-income settings. Yet evidence consistently shows that vulnerable populations face compounding risks during evacuations, including care disruptions and inadequate shelter conditions [14, 15, 17, 41]. Flexible Surge Capacity (FSC) represents a community-based disaster management approach that mobilizes and repurposes existing medical and non-medical resources into Alternative Care Facilities (ACFs) to meet emergent health and social needs [42–44]. In the context of evacuation, ACFs offer a scalable solution to accommodate displaced individuals–particularly those with functional, medical, or psychosocial needs–when conventional care structures are insufficient or inaccessible. Despite their relevance, the application of FSC and ACFs in flood-related evacuations remains underexplored in both theory and practice, especially regarding their potential role in supporting vulnerable groups.

The present review seeks to address these gaps by focusing on a distinct and underexamined intersection of vulnerable groups (population), evacuation and vulnerability management (concept), and flood-induced disasters in HICs (context). By applying evolving conceptual models of vulnerability to the concrete operational challenge of evacuation management, this review offers a practice-oriented, phase-specific perspective that advances inclusive and actionable disaster management strategies. Building on the insights of recent reviews, it contributes to bridging the gap between theoretical advancements and real-world implementation. Given the complexity and fragmented state of existing evidence, a scoping review is the most suitable approach to map the literature, clarify definitions, and identify knowledge gaps. This review aims to explore how vulnerability is defined, addressed, and operationalized in the context of flood-induced disaster evacuations in HICs. Specifically, it seeks to: (i) map definitions and assessment approaches to vulnerability; (ii) identify population groups deemed vulnerable during flood-induced disasters, and their specific evacuation needs; (iii) examine evacuation and sheltering strategies–particularly the implementation of FSC through the use of ACFs as a scalable and community-based model; and (iv) synthesize challenges and promising practices in planning and delivering evacuation for vulnerable individuals during flood-induced disasters. The broader aim of this review is to inform more inclusive and effective flood-induced disaster management, with a particular focus on improving evacuation and protection of vulnerable individuals.

## Methods

### Study design

A systematic scoping review was conducted in adherence to the Preferred Reporting Items for Systematic Reviews and Meta-Analyses extension for Scoping Reviews (PRISMA-ScR) guidelines [45] and the Joanna Briggs Institute (JBI) Manual for Evidence Synthesis framework [46].

### Search strategy

A comprehensive literature search was conducted on March 3, 2025, using Web of Science, PubMed, and Scopus. These databases were selected for their complementary strengths: PubMed provides precise coverage of biomedical literature, Scopus offers broad multidisciplinary coverage and strong citation analysis, while Web of Science is known for its high-quality content and in-depth citation tracking. Their combined use ensures a comprehensive and robust literature review. Additionally, a manual search was performed to identify relevant studies that may not have been retrieved through database search. The search strategy (Supplementary Material I) was designed to be comprehensive and specific to the review’s objective: examine how vulnerability is conceptualized, managed, and put into practice within the context of flood-induced disaster evacuations in HICs. To construct search strings, three primary concepts were considered: (1) flood-induced disasters, (2) vulnerability, and (3) evacuation. Each concept was expanded into a set of keywords, reflecting the different dimensions of each topic. The strings were tailored to each database’s syntax and incorporated Boolean logic and truncations.

### Eligibility criteria

Studies retrieved were included based on the following criteria: (a) type of study: only primary research articles were considered (i.e., reviews, letters and conference papers were excluded); (b) study focus: studies had to investigate the evacuation process of vulnerable groups during flood-induced disasters or provide information on the characteristics and impacts of flood-induced disasters on affected vulnerable populations; (c) publication date and language: articles published after January 1, 2014, onward and written in English; (d) study context: studies conducted in HICs, as classified by the World Bank Group’s country classification by income level [47] (Supplementary Material II). The decision to limit the scope to HICs was based on the premise that countries with similar income levels tend to have comparable disaster management practices, capacities, and resources. By ensuring that included studies originate from HICs, the findings of this review remain homogeneous and generalizable within contexts sharing similar disaster management characteristics and capabilities.

### Study selection process

The study selection process was conducted by two researchers (CDP and AMS). All citations retrieved from the database searches were systematically compiled into a Google Sheet document, where duplicates were removed. Titles and abstracts were independently screened for eligibility on the predefined inclusion criteria. Articles meeting eligibility requirements underwent a full-text screening for final inclusion in the study.

### Data extraction and synthesis

Relevant information from included studies was systematically charted using a Google Sheet document following a deductive approach. Data were thematically extracted to align with the study’s objective. A structured extraction table (Supplementary Material III) was employed to collect information on key aspects, including flood-induced disaster type, study characteristics, evacuation process, vulnerability definition used, identified vulnerable groups, and their specific characteristics and needs, in line with the objective of the review. The extracted data were then synthesized according to predefined categories within the extraction table, which facilitated the systematic collection of information into sub-themes focusing on the characteristics, impacts and implications for vulnerable groups’ evacuation during flood-induced disasters.

## Results

**Fig. 1:**
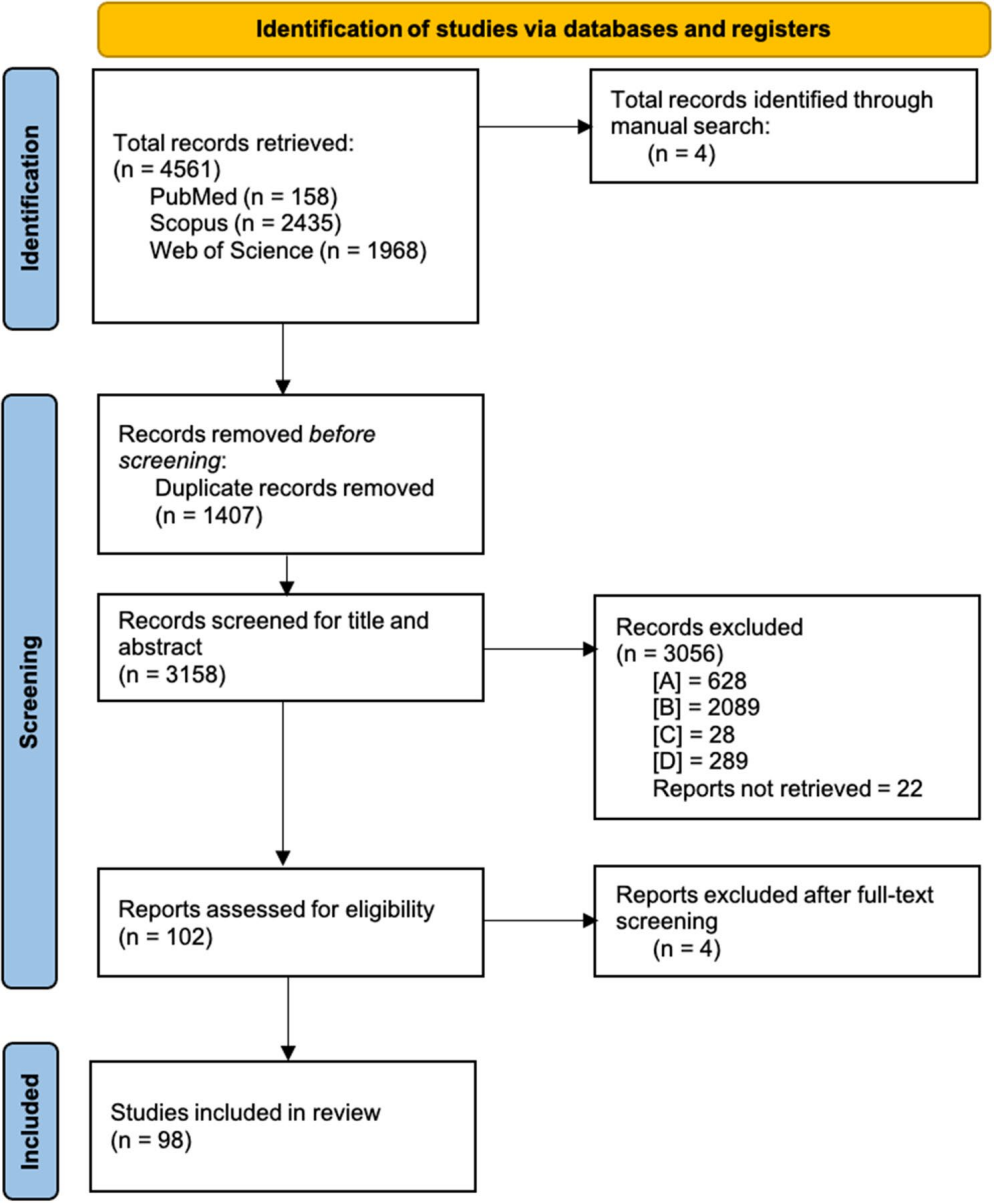
PRISMA 2020 flow diagram [145]

**Fig. 2:**
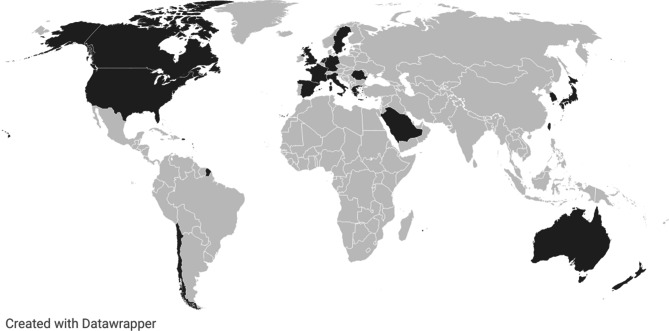
Countries included in the review

A total of 4561 studies were identified through database searches. After removing 1407 duplicates, 3158 studies remained for screening. Following title and abstract screening, 3056 studies were excluded based on the eligibility criteria. A subsequent full-text screening led to the exclusion of four additional studies, resulting in a final inclusion of 98 studies (Fig. 1). The included studies were published between 2014 and 2025, with 2020 being the most represented year. Among these, 61 employed a quantitative methodology, 26 used qualitative methods and 11 adopted a mixed-methods approach. The geographical distribution of the included studies shows that all were conducted in HICs (Fig. 2), with the majority originating from the United States (43) and Japan (14). Several European countries were also represented (17). Additional studies came from South Korea (4) and Australia (3). Regarding hazard type, floods were the most frequently studied event (65), followed by hurricanes (28), tsunamis (10), typhoons (5) and cyclones (1). A summary of the characteristics of included studies is presented in Table 1.

**Table 1:** Characteristics of included studies

Study characteristics	Number	References
**Year of publication**		
2014	4	[48–51]
2015	4	[52–55]
2016	9	[56–64]
2017	7	[65–71]
2018	7	[72–78]
2019	7	[79–85]
2020	13	[86–98]
2021	12	[99–110]
2022	10	[111–120]
2023	12	[121–132]
2024	9	[133–141]
2025	4	[17, 142–144]
**Methodology**		
***Quantitative***		
Secondary data analysis	26	[60, 61, 65, 66, 68, 83–85, 90, 91, 93, 96, 98, 102–104, 117, 121, 123, 126, 131, 133–136, 138]
Simulation modeling and case study	23	[51–54, 67, 78, 79, 81, 97, 100, 106, 109, 113, 118, 119, 122, 124, 125, 127, 130, 139, 142, 143]
Survey	8	[49, 55, 70, 82, 88, 101, 128, 141]
Other	4	[57, 59, 87, 132]
***Qualitative***		
Interviews and Focus Group Discussions	15	[17, 56, 58, 62, 63, 69, 71–74, 86, 92, 105, 107, 112]
Descriptive and evaluative studies	5	[48, 77, 94, 116, 129]
Participatory art method	1	[99]
Other	5	[75, 80, 89, 120, 144]
***Mixed-methods***		
Interviews and Focus Group Discussions + Secondary data analysis	3	[76, 110, 140]
Interviews and Focus Group Discussions + Survey	4	[50, 95, 131, 137]
Secondary data analysis + Simulation modeling and case study	2	[114, 115]
Survey + Descriptive and evaluative studies	2	[108, 111]
**Geographical setting**		
**Worldwide**	3	[51, 114, 131]
***North America***		
United States	43	[48, 49, 52, 56–59, 61, 63, 65, 66, 68, 72, 73, 76, 80, 81, 83, 86, 88, 96, 101–103, 105, 112, 115, 117, 122, 124–128, 130, 132, 133, 135, 136, 138, 139, 142, 143]
Canada	6	[54, 78, 82, 92, 107, 137]
Puerto Rico	1	[104]
***South America***		
Chile	3	[75, 79, 84, 91]
***Europe***		
France	2	[87, 141]
Germany	2	[64, 144]
Greece	1	[123]
Italy	1	[17]
Netherlands	3	[51, 53, 85]
Romania	2	[99, 113]
Spain	1	[134]
Sweden	1	[70]
UK	4	[67, 74, 90, 97]
***Asia***		
Japan	14	[50, 60, 69, 77, 89, 93, 94, 108–110, 118, 120, 121, 140]
South Korea	4	[55, 98, 100, 119]
Taiwan	2	[106, 129]
Saudi Arabia	1	[95]
***Oceania***		
Australia	3	[62, 111, 116]
New Zealand	1	[71]
**Type of flood-induced disaster**		
Floods	65	[17, 50–56, 61, 62, 64, 66–72, 74, 75, 77, 82–85, 87, 89, 90, 92, 93, 95–100, 106, 107, 110–114, 116, 119–123, 125, 127, 128, 131–141, 143, 144]
Hurricanes	28	[48, 49, 52, 56–59, 63, 65, 73, 76, 80, 86, 88, 101–105, 114, 117, 124, 126, 130, 132, 142] [66]
Tsunamis	10	[52, 60, 78, 79, 81, 91, 108, 109, 118, 123]
Typhoons	5	[56, 89, 94, 119, 129]
Cyclones	1	[111]

### Definitions and measures of vulnerability

Two studies addressed the challenge of defining vulnerability universally [93, 131], with one identifying two main dimensions: physical and social [93]. The physical dimension, distinct from biological aspects, includes geographical, locational, and structural factors [93, 136, 138]. The social dimension [59, 65, 73, 74, 85, 88, 89, 93, 102, 103, 107, 115, 126, 127, 131, 133, 136, 138] encompasses inequalities shaping disaster susceptibility [73, 93], incorporating biophysical vulnerabilities [59, 93], social determinants [102, 107, 126, 133, 136], and broader political, institutional, economic, and ideological factors [107, 133]. These inequalities affect disaster sensitivity, survival capacity [126, 136], extent of harm [107, 133], health outcomes, healthcare access [126], and post-disaster needs [102]. Social vulnerability [54, 85, 102, 115, 127, 139, 141] refers to social, economic, demographic, geographical inequalities [54, 85, 102, 136], and susceptibility [54, 85, 136], disproportionately impacting certain individuals [115]. Cumulative vulnerability [74] and multidimensional flood vulnerability were also noted [50]. Several studies highlighted vulnerability indexes, as tools to assess social, economic, and environmental risk factors. The most frequently cited indices are summarized in Table 2 and detailed in Supplementary Material IV.

**Table 2:** Vulnerability indexes cited in the included papers

Index	References
Social Vulnerability Index (SoVI)	[53, 54, 65, 83, 88, 91, 96, 122, 132, 136]
CDC Social Vulnerability Index (SVI)	[96, 103, 122, 126, 133, 136]
Social Vulnerability Score (SVS)	[125]
US Social Vulnerability Index	[73]
Climate Displacement and Socio-Vulnerability (CDSV) score	[132]
Hazard Exposure Vulnerability Index (HazVI)	[65]
Built Environment Vulnerability Index (BEVI)	[65]
Hazards Level Index (HLI)	[83]
Community Disaster Resilience Index (CDRI)	[96]
Community Level Index (CLI)	[83]
Nursing Home Level Index (NHLI)	[83]
Social Flood Vulnerability Index (SFVI)	[90]
Acuindex	[117]
Flood Vulnerability Index	[117]
Social Flood Vulnerability Index (SFVI)	[90]
Coastal City Flood Vulnerability Index (CCFVI)	[132]
Extreme Inherent Vulnerability (VIE) Index	[87]
Tsunami Risk Index	[79]
Social Vulnerability for Evacuation Assistance Index (SVEAI)	[54]
Evacuation Vulnerability Index	[65]
Resilience Capacity Index (RCI)	[96]
Response Time by Social Vulnerability Index (ReTSVI)	[84]

### The link between flood-induced disasters, vulnerability and evacuations

The analysis identified key links between flood-induced disasters, vulnerability, and evacuations, showing that evacuations can alter vulnerability dynamics, either creating new risks or worsening existing ones. Several barriers to safe and effective evacuation were noted [51, 54, 65, 110, 115], including age [51], minority status, gender [51], mobility limitations [51, 110], financial constraints [51], discrimination, distrust in aid providers [115], lack of private vehicles or insufficient public transportation [65]. Assessment tools used to analyze the vulnerability-evacuation interplay were cited, including the Evacuation Vulnerability Index [65] and the Social Vulnerability for Evacuation Assistance Index (SVEAI) [54].

### Vulnerability factors during flood-induced disasters

Several vulnerability factors within the context of flood-induced disasters and evacuations were identified and summarized in Table 3.

**Table 3:** Vulnerability factors cited in the included papers

Vulnerability factors	Number	References
***Social and demographic***		
Race	8	[54, 65, 88, 102, 114, 115, 132, 136]
Cultural dynamics	1	[88]
Ethnicity and minority status	17	[51, 53, 68, 84, 88, 91, 96, 102–104, 107, 115, 118, 126, 132, 135, 136]
Immigration status*	1	[91]
Foreigner status*	4	[83, 85, 110, 118]
Religious minorities status	3	[95, 114, 115]
Language proficiency barriers	5	[65, 102, 103, 118, 136]
Lack of social ties and networks	2	[107, 126]
***Gender and family structure***		
Gender disparities	12	[51, 54, 65, 84, 85, 88, 91, 102, 107, 114, 115, 121]
Family structure	8	[53, 83, 85, 91, 103, 115, 132, 136]
Divorced and single-parent status	2	[84, 136]
Female head of the house status	2	[91, 96]
***Age and health***		
Older age	12	[51, 59, 84, 85, 91, 103, 110, 115, 118, 126, 135, 136]
Younger age	8	[51, 85, 91, 103, 110, 126, 132, 136]
Age in general	13	[53, 54, 64, 65, 83, 88, 95, 102, 107, 114, 115, 121, 136]
Having special needs and being medically fragile	3	[51, 59, 110]
Having a disability	9	[51, 65, 91, 103, 115, 121, 126, 135, 136]
Having a chronic illness and/or comorbidities	4	[103, 104, 115, 132]
Residing in a nursing home	1	[83]
Lacking health insurance	5	[65, 84, 96, 104, 118]
Being pregnant	1	[110]
Having a caregiving role	1	[115]
***Economic and educational***		
Low-income	24	[51, 53, 54, 59, 64, 65, 68, 83, 85, 88, 91, 102–104, 107, 110, 114, 115, 118, 121, 126, 132, 135, 136]
Unemployment	11	[65, 83, 85, 91, 95, 103, 114, 115, 118, 132, 136]
Occupation type	4	[84, 88, 91, 136].
Low educational level	14	[65, 83–85, 91, 95, 102, 103, 114, 118, 121, 126, 132, 136]
Limited hazard knowledge and disaster experience	1	[107]
***Housing and infrastructure***		
Tenancy status	9	[65, 83–85, 88, 95, 96, 118, 136]
Built environment	1	[88]
Household density	3	[83, 84, 136]
House value	4	[65, 83, 85, 96]
House material	1	[91]
Construction year	2	[53, 85]
Housing type (mobile homes, multi-unit structures)		[65, 83, 91, 96, 103, 136]
Living in crowded conditions	7	[84, 85, 91, 96, 103, 118, 136]
Living in informal settlements	1	[84]
Living in areas with crime proliferation	1	[126]
Homelessness	2	[57, 103]
***Access and mobility***		
Lack of basic services	1	[91]
Infrastructure dependence	1	[88]
Lack of vehicles	7	[65, 83, 85, 103, 132, 135, 136]
Lack of telephone ownership	1	[65]
Lack of access to social networks and community organizations	1	[95]
Lack of access to information	2	[51, 95]
Lack of access to medical services	3	[59, 85, 104]
Distance from essential facilities	1	[85]

*Immigration status denotes long-term or permanent residents with varying legal statuses, whereas foreigner status refers to short-term non-residents such as tourists or temporary visitors

### Vulnerable groups during flood-induced disasters

**Fig. 3:**
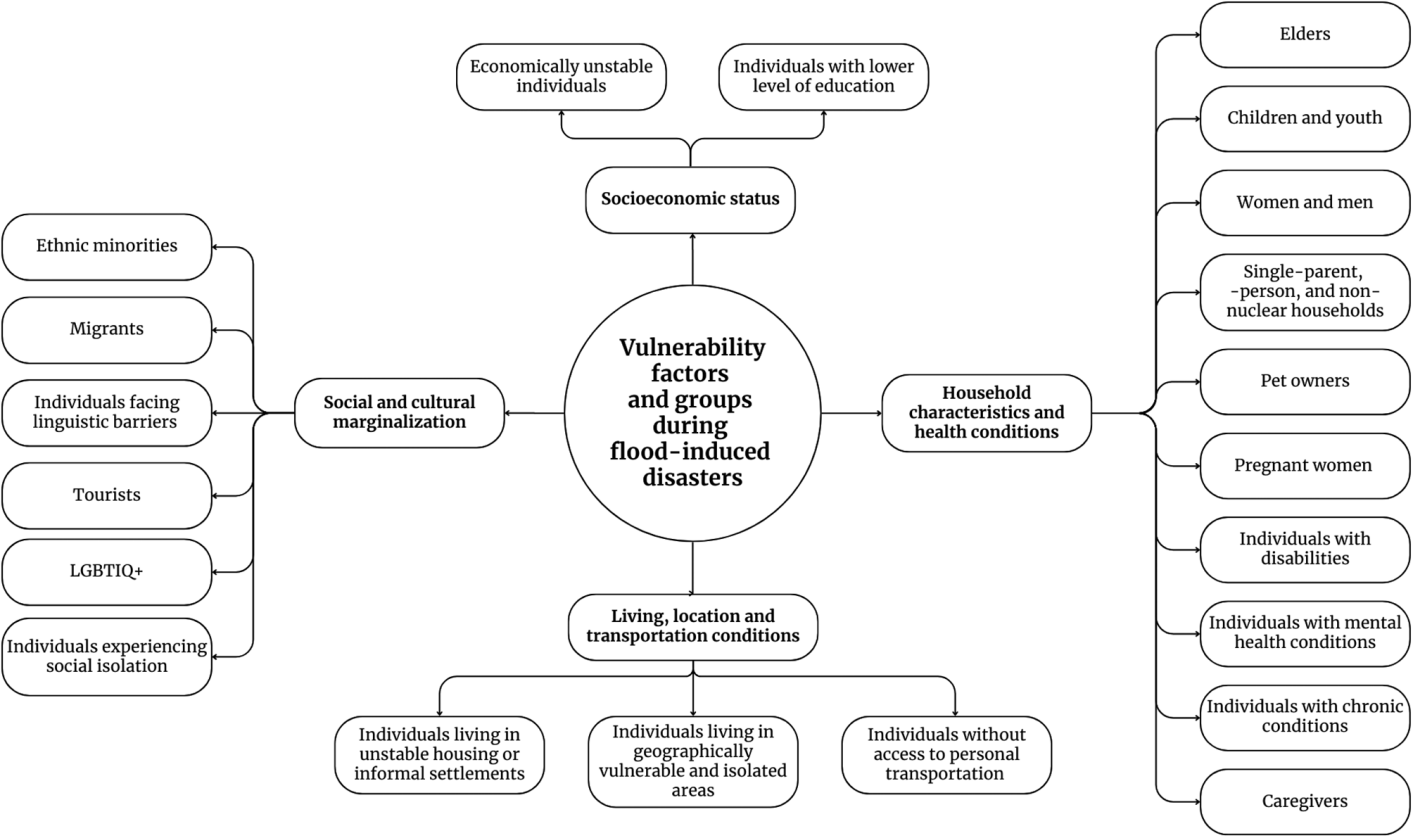
Identified vulnerability factors and vulnerable groups during flood-induced disasters

The synthesis identified several vulnerable groups–individuals reportedly more affected by flood-induced disasters due to one or more vulnerability factors (Table 4 and Fig. 3).

**Table 4:** Identified vulnerable groups during flood-induced disasters

Vulnerable group	Number	References
***Household characteristics and health conditions***		
Elders	70	[17, 51–55, 57, 59, 61, 63, 65–67, 69–73, 76–79, 81–85, 87, 88, 90, 91, 93–98, 100–103, 105–108, 110, 113–118, 121, 122, 126–128, 130, 131, 133–139, 141–144]
Children and youth	36	[17, 48, 51–54, 59, 63, 69, 70, 73, 74, 79, 83, 84, 91, 95–104, 108, 113, 124–126, 128, 132–134, 136, 143]
Women and men	20	[49, 51, 57, 63, 76, 83–86, 91, 93, 96, 102, 107, 114, 115, 121, 131, 137, 138]
Single-parent, -person, and non-nuclear households	13	[49, 53, 91, 92, 103, 107, 128, 131–133, 136, 137, 143]
Pet owners	7	[58, 62, 88, 92, 105, 128, 131]
Pregnant women	3	[69, 108, 110]
Individuals with disabilities	42	[17, 49, 51, 54, 59, 61, 63, 65, 69, 70, 73, 76–79, 83, 84, 90, 91, 94, 97, 101, 105, 106, 108, 110, 111, 113, 115, 117, 118, 121, 122, 126, 128, 131, 133, 135–137, 141]
Individuals with mental health conditions	6	[51, 59, 73, 90, 117, 137]
Individuals with chronic conditions	35	[17, 49, 54, 59, 61–63, 70, 72, 73, 76, 79, 83, 90, 91, 94, 97, 101, 103–105, 107–110, 115, 117, 118, 122, 126, 128, 131, 132, 137, 140]
Caregivers	8	[17, 49, 72, 95, 105, 111, 115, 117]
***Socioeconomic status***		
Economically unstable individuals	42	[49, 51, 53, 59, 61, 66, 68, 74, 82–85, 88, 91, 93, 95–97, 101–103, 110, 111, 114, 115, 118, 121, 122, 125–128, 131–133, 135–139, 141, 142]
Individuals with lower levels of education	19	[63, 82–84, 91, 95, 101–103, 121, 127, 128, 131–133, 136, 137, 141, 142]
***Social and cultural marginalization***		
Ethnic minorities	26	[51, 66, 68, 73, 76, 84, 86, 91, 95–97, 102, 103, 105, 107, 114, 118, 128, 132, 133, 135–138, 142, 143]
Migrants	9	[53, 91, 95, 102, 105, 112, 127, 131, 136]
Individuals facing linguistic barriers	10	[17, 56, 61, 80, 91, 102, 112, 131, 133, 141]
Tourists	4	[69, 78, 81, 88]
LGBTIQ+	2	[71, 115]
Individuals experiencing social isolation	3	[49, 88, 114]
***Living, location and transportation conditions***		
Individuals living in unstable housing or informal settlements	25	[49, 53, 57, 61, 73, 74, 84, 85, 87, 88, 91, 94–96, 99, 102, 103, 125, 128, 131, 133, 136–138, 141]
Individuals living in geographically vulnerable and isolated areas	25	[17, 49, 51, 59, 79, 80, 85, 86, 88, 91, 93, 95–97, 112, 114, 119, 122, 126, 131, 134, 135, 141–143]
Individuals without access to personal transportation	16	[54, 61, 65, 66, 85, 102, 105, 126, 127, 132, 133, 135, 136, 141–143]

#### Household characteristics and health conditions

##### Elders

The age threshold for defining elders varied: 60+ [100, 128], 65+ [53, 61, 65, 72, 79, 85, 90, 91, 93–96, 98, 101, 102, 116, 128, 130, 136, 137, 142], 70–79 [82], and 75+ years [93]. Their vulnerability was described as stemming from physical limitations (mobility [61, 72, 77, 83, 93, 101, 102, 117] and sensory [72, 83, 117] impairments, age-related disabilities [72, 102, 117], cognitive decline [72, 77, 83, 101, 102, 117], including Alzheimer’s Disease and Related Dementias [71, 117]), comorbidities, chronic conditions [61, 72, 101, 117, 133], and socioeconomic factors (fixed income, poverty [61, 72, 83], social isolation, rural residency [53, 61, 102, 117]). Subgroups include homeless elders [57] and retirees in flood-prone areas [88, 91]. Lower education levels were reported to limit their ability to understand and act on emergency instructions [72]. Studies classified elders as housebound, bed-bound [110], or nursing home residents [90]. It was reported that ambulance demand for medically fragile elders increased during floods [90], and that they are particularly affected by medical service disruption during flood-induced disasters [96, 122], with ambulance coverage gaps reported in England [97]. Elders were often described as among the least prepared for flood-induced disasters [85], and as facing greater difficulty to respond, adapt, recover [59, 82, 85, 117], and at higher exposure to flooding, storm surges, and medical supply shortages [117]. There are reports of elderly people becoming trapped for days on higher floors during flood-induced disasters [73]. Morbidity rates were higher during flood-induced disasters [59, 72, 76, 83, 87, 101, 102, 117, 144], comprising acute injuries [59], emergency department (ED) visits [102], mental health issues [83, 87], and hospitalization during evacuations [101]. Their death rates were higher [72, 76, 102] during tsunamis [79], floods [61, 96], and hurricanes [83, 101], with nursing home residents disproportionately affected [83, 117]. Evacuation challenges during flood-induced disasters for elders were reported as significant [93, 94, 101], as many were transit-dependent [65], and relied on family for assistance [63, 72, 95]. Reduced mobility and slower evacuation speeds hinder escape [53, 61, 78, 79, 94, 106, 108], particularly for nursing home and aged care facility residents, hospital patients, and those living alone, who faced greater difficulties if geographically isolated [83, 108, 116]. Difficulties in reaching shelters were reported for elders [93], with distance to evacuation points as a major barrier [65]. Evacuation decisions were reported to be influenced by physical and cognitive impairments and socioeconomic constraints [128]. Studies also highlighted how they often ignored evacuation orders, struggled to comply with procedures [61, 83], and were less willing to evacuate than other groups [61, 83], with some sheltering-in-place [63, 101, 114]. During hurricanes, reluctance was linked to needing assistance [128], while during pandemic-compounded disasters, reported concerns over social distancing in shelters deterred them [101, 128]. Elderly evacuees were found to require specialized care and family support during evacuation [95]. In Florida, special needs shelters provided medical care for these individuals [130]. A study on post-hurricane needs in the US found that shelter needs increased by 0.033 for every 1% rise in the 65+ population (B = 0.033, *p* < 0.001) [102]. After Michigan’s 2020 floods, older evacuees returned home earlier than younger ones, with a 1-year increase in age reducing the odds of staying in a shelter by 3% (*p* < 0.1), possibly due to a preference for familiar environments or difficulties adapting to shelters [128].

##### Children and youth

The age threshold for defining children varied: infants [69, 102], under 1 year [84], 0–4 years [98], under 5 [96, 102], under 6 [79], 7–17 years [124], under 14 [91, 136], under 15 [100] under 17 [132], and under 18 [133]. This group was reported to experience significant vulnerabilities, multifaceted needs [51, 79, 97, 98, 100], and high susceptibility to floods [70, 97]. Children and youth under 18 were found to face heightened risk due to their inability to evacuate and to seek help independently [98], limited capacity to cope with flood-induced disasters [133], and exacerbation of pre-existing challenges [74]. Homeless children were reported to encounter additional risks, including physical harm, psychological distress, and disrupted education during flood-induced disasters [125]. Households with younger children were described as more common in rural areas compared to other areas [53]. Medically fragile children, such as those with neurodevelopmental disorders, were described as facing unique evacuation and sheltering challenges during flood-induced disasters, as environmental disruptions can worsen behavioral symptoms and lead parents to avoid shelters to prevent stigma [108]. Children were found to have increased emergency needs [102] and to be disproportionately affected by health risks during flood-induced disasters, including infections [104]. After Hurricane Katrina, 75,000 infants were exposed to contaminants and suffered from healthcare and medical disruptions [102]. Middle school students experienced significant psychological distress, somatic complaints, and increased sedentary behavior [124]. Young children under 5 had higher mortality rates during tsunamis [79] and floods [96]. Evacuations disrupt children’s routines, leading to frustration and disillusionment [74]. Households with children in Florida were reported to be 6.6 times more likely to evacuate during hurricanes compared to other households [63], but young children’s dependence and reduced mobility were reported to complicate evacuation [69, 79, 91, 98]. In the US, post-hurricane data showed that for every 1% rise in the under-5 population, shelter needs increased by 0.034 [98]. During the 2020 Michigan floods, households with children tended to stay in shelters for less than four days [128].

##### Women and men

Women were consistently reported to be disproportionately vulnerable to floods [51, 76, 84, 86, 91, 102, 114, 121] due to intersecting socioeconomic factors, societal status, and caregiving roles [85, 91, 102, 114, 121]. Caregiving roles were described as significantly increasing their exposure [91, 121] and as contributing to heightened chronic anxiety regarding health and family well-being [102]. Female-headed households were identified as facing heightened vulnerability [91], as illustrated by a single mother’s account of acute stress, fear, and logistical challenges during a flood [107]. Post-disaster studies found that women were more likely than men to feel insecure [102]. After Hurricane Katrina, women constituted 80% of those remaining in New Orleans after the evacuation orders [121], and, within a year, gender-based violence was reported to have increased in Mississippi trailer parks [102]. Floods were found to reduce women’s life expectancy more than men’s [102], although this was influenced by socioeconomic status [121]. Similar trends were observed in the 2011 Tohoku earthquake and tsunami [76]. Post-Hurricane Sandy data showed that women were more frequently diagnosed with homelessness and inadequate housing in EDs [57]. Women’s evacuation decisions were reported to be influenced by financial burdens, potential job loss, and fear of discrimination [114]. In the Netherlands, lower female participation in full-time work was linked to increased flood-induced disaster vulnerability [85]. US post-hurricane data showed that for every 1% increase in the female population, per capita reported emergency needs rose by 0.071 and food needs by 0.046, while, shelter needs were not statistically significant, possibly due to women’s reluctance toward unfamiliar accommodations [102].

Men were described as exhibiting distinct vulnerabilities during flood-induced disasters [49, 96, 102, 121]. They were more likely to engage in hazardous behaviors, such as attempting to drive through floodwaters, which significantly increased their fatality risk compared to women [96]. In the US, most flood-related deaths involved men drowning in vehicles [96]. Gender differences in flood fatalities were found to be age-dependent, with men predominantly represented in the 30–49 age group, while women accounted for more deaths over 65 than men [121]. Men were also reported to be less likely than women to evacuate: for instance, during Hurricane Irene, male residents in the US were 0.708 times less likely to evacuate than women [49]. A survey in Nagasaki City, Japan found that during floods, 8.3% of men resisted evacuation compared to 1.3% of women and that family evacuation decisions often depended on the husband, who prioritized evacuating senior and very young children before deciding for himself [121]. Men were also described as perceiving requesting disaster assistance, particularly financial aid, as undermining their household role [102].

##### Single-parent, -person and non-nuclear households

These households were reported to face heightened flood-related challenges due to financial constraints, work obligations, and caregiving duties [53, 91, 107, 136]. Women-led households, where caregivers had to balance family safety with essential tasks, were described as experiencing greater exposure to flood-induced disasters impacts [91]. A qualitative study illustrated this through the account of a single mother who reported feeling overwhelmed by the dual burden of managing the flood alone and ensuring her children’s safety [107]. In urban Rotterdam and Dordrecht, high levels of social vulnerability were found to be associated with low-income, minority status and a high prevalence of single-parent households [53]. Medically fragile single-parent households were identified as being 0.454 times less likely to evacuate than other households, with no other factor influencing evacuation likelihood as strongly as this household characteristics [49].

##### Pet owners

Flood-induced disaster management was reported to often overlook the needs of pet owners and their animals [58, 62, 92, 105, 128]. Pet owners were found to require both immediate pet-friendly shelters and longer-term housing solutions [92]. They were described as more hesitant to evacuate [105], with studies showing lower evacuation rates among households with pets compared to households without pets. In the US, pet-owning households were observed to evacuate less frequently than those without pets [128], and 20% of individuals who refused to evacuate cited pets as their primary reason [88]. In North Carolina, pet owners were found to be 52% more likely to remain at home due to logistical and financial constraints compared to households without pets [58]. Australian evacuees reported being unwilling to leave if their pets could not accompany them, even in life-threatening situations [62]. Pet owners were also found to stay in shelters for shorter periods [128] and often return prematurely to disaster zones to rescue their animals, putting themselves and responders at risk [58, 62]. Losing a pet was described as having significant psychological effects, and uncontrolled animals in flood-affected areas was linked to environmental contamination and zoonotic disease transmission [58]. After the 2013 Alberta floods, demand for pet-friendly rental housing was reported to have exceeded supply, with fewer than 10% of advertised rental units allowing pets and imposing higher rents and pet size and breed restrictions [92].

##### Pregnant women

In Japan, pregnant women were officially recognized as a priority group requiring additional disaster evacuation assistance [108]. They are classified under the government’s People Requiring Assistance During a Disaster and Persons Requiring Special Consideration frameworks, which acknowledged their need for support in taking protective actions and evacuating safely during flood-induced disasters [69].

##### Individuals with disabilities

Disability definitions varied, encompassing physical, cognitive, and sensory impairments. One study cited the American Community Survey’s six categories of disability: hearing, vision, cognitive, ambulatory, self-care, and independent living disabilities [65]. Other studies defined disability more broadly as medically fragile individuals, those with physical or mental challenges, and nursing home residents requiring continuous care [49, 90, 133]. Individuals with disabilities were further classified as housebound or bed-bound [110], and as belonging to other vulnerable groups simultaneously [51]. Their vulnerability was reported to stem from pre-existing conditions and situational factors [70, 73, 84, 91, 97, 111, 122, 126, 128, 131, 135], increasing risks during floods [84, 97, 111, 122, 128, 131, 135] and hurricanes [126]. They were described as facing societal invisibility [91], higher mortality, injury risks, loss of property, and disrupted support systems during flood-induced disasters [111]. In rural Australia, individuals with disabilities were found to be three times more likely to develop Post-Traumatic Stress Disorder (PTSD) and 1.76 times more likely to remain in distress following a flood than individuals without disabilities [111]. Renters with disabilities in flood-prone areas were identified as having the fewest resources for coping and recovery [111]. They also faced barriers to accessing medical care, evacuation and sheltering, extended recovery times, and greater post-disaster care needs [111, 122]. In rural Australia, individuals with disabilities were found to be significantly more likely to experience disrupted access to healthcare (OR: 3.98, 95% CI 2.82–5.60) and food supplies (OR: 2.06, 95% CI 1.45–2.91) compared to other groups [111]. Many reported that available recovery assistance was inaccessible [111]. Evacuation presented major challenges for individuals with disabilities during flood-induced disasters [49, 61, 63, 65, 69, 73, 79, 101, 105, 106, 108, 118, 128, 133, 135] due to physical impairments, logistical barriers, and emotional distress [63, 105, 128]. In rural Australia, individuals with disabilities and their caregivers, compared to other groups, were twice as likely to have their homes flooded (OR: 2.41 and 1.76, respectively), nearly four times more likely to be displaced for over six months (OR: 3.78, 95%CI 2.18 to 6.55), and twice as likely to require evacuation while facing significant barriers (OR: 2.46, 95% CI 1.71 to 3.54) [111]. Physical impairments were reported to slow evacuations, while cognitive disabilities increased stress and dependency on caregivers [61, 106, 133]. During Hurricane Sandy, nearly 700 inoperable elevators left many residents with disabilities trapped in high-rise buildings for extended periods [73]. Lack of accessible infrastructure and difficulties transporting medical equipment further exacerbated evacuation challenges, emphasizing the need for specialized evacuation protocols [133]. Being transit-dependent made inaccessible pickup points during evacuation critical [65]. Recognizing these barriers, Japan has formally categorized individuals with disabilities as a priority evacuation group [69, 108] and integrated them into national disaster guidelines [108]. Japan also developed evacuation registries and plans accommodating different evacuation speeds based on physical capabilities [118]. Communication barriers posed additional risks: rural Australian respondents reported difficulty interpreting evacuation orders due to technical language and unclear instructions, while deaf individuals lacked access to life-saving information when evacuation messages were delivered orally without sign language [111]. During Hurricane Katrina, inadequate communication strategies were reported to have disproportionately affected those with sensory disabilities [49]. A Florida study on special needs shelters found that while 7.1% of respondents required these shelters, only 1.2% applied to determine eligibility [101]. Many individuals with disabilities chose not to evacuate due to inaccessible shelter conditions [49, 101, 106, 133, 135], lack of disability-inclusive evacuation plans [69], difficulty adapting to unfamiliar environments–particularly for the visually impaired–and challenges faced by households with children with Attention-Deficit/Hyperactivity Disorder in adjusting to evacuation centers [69]. Even in areas with high shelter density, individuals with disabilities were reported to face barriers to access [135]. Those who do evacuate were found to be more likely to remain in shelters for extended periods, often a week or longer compared to other evacuees [128].

##### Individuals with mental health conditions

Nursing home residents with mental health conditions were identified as among the most at-risk during flood-induced disasters. One study found that 78% of nursing home residents had abnormal mental capacity and required 24-hour personal care, making them highly dependent [90]. These individuals faced significant evacuation challenges, which further increased their vulnerability [51]. A major barrier to evacuation was reported to be the heightened risk associated with relocation. Nursing homes with a higher percentage of residents receiving anti-anxiety medications were less likely to evacuate compared to other facilities, as these individuals were particularly vulnerable to the psychological and physical strain of relocation [117]. Evacuation process, combined with exposure to unfamiliar environments, discontinuity of care, and separation from routine, was found to exacerbate anxiety, dementia, depression, and PTSD, worsening overall health outcomes and increasing caregiver strain [117].

##### Individuals with chronic conditions

Chronic conditions were reported to limit individuals’ ability to adapt to rapidly changing environments during flood-induced disasters [49, 59, 128]. Those with chronic conditions often relied on medical regimens and life-sustaining devices, while also facing sensory, cognitive, and mobility impairments [59, 72]. Symptoms such as pain, fatigue, breathing difficulties, and muscular weakness further exacerbated vulnerability [70]. Frequently cited non-communicable diseases (NCDs) included diabetes [49, 62, 101, 104, 132], hypertension [49, 104], high blood pressure [49], cardiovascular diseases [49, 62, 104, 122], congestive heart failure [70], respiratory illnesses [62, 117], such as chronic obstructive pulmonary disease (COPD) [70, 118], asthma [101, 132], and interstitial pneumonia [118], renal diseases [62, 117], cancer [62], Alzheimer’s Disease and Related Dementias [72], and immunocompromised states [101]. Many depended on dialysis [62], oxygen therapy and other electrically powered medical devices [101, 104, 118]. Advance care home [70] and nursing home patients also required specialized care, including treatment for pressure ulcers, and urinary tract infections, as well as tube feeding and respiratory support [83]. Individuals with chronic conditions and comorbidities were identified as at high risk during flood-induced disasters [59, 62, 70, 72, 97, 101, 104, 117, 122, 126, 128, 132], with pre-existing health needs amplifying risks [97, 128] during floods [72, 97, 122, 128, 132]. Healthcare disruptions were commonly reported [104] during floods [107, 122], storms [59], hurricanes [104, 126], and cyclones [62]. After Hurricane Maria in Puerto Rico, primary care clinic closures and shortages of medical professionals created a gap in chronic disease management [104]. Patients with chronic diseases frequently struggled to obtain medications, routine care, and support during flood-induced disasters [59, 62, 104]. Power outages severely impacted NCD management, jeopardizing insulin refrigeration, disabling oxygen concentrators and dialysis machines, leading to increased reliance on ambulance services [62] and compromising patients’ survival, as seen in Sweden, where 6.4% of advance care home patients were reported to experience complications within the first three hours [70]. Damaged sanitation infrastructure heightened infection risks for NCD patients [62]. Staffing shortages at dialysis centers delayed critical treatment and reduce follow-up care [62]. Many individuals with NCDs forgot medications during evacuation, worsening their conditions [62]. Following Hurricane Maria, individuals with chronic illnesses reported difficulty in managing their conditions, increased infections, and musculoskeletal complaints [104]. Older adults with chronic conditions had the highest disaster-related mortality rates [72]. Disruptions to medical services and road closures during flood-induced disasters resulted in life-threatening consequences for individuals with chronic conditions–such as cancer, diabetes, hypertension, heart failure, and respiratory diseases–by delaying access to critical care, dialysis, opioid treatments, and medications, thereby increasing the risk of cardiac arrest and severe complications for transplant recipients and those undergoing dialysis [59, 62, 104, 122]. Evacuation during flood-induced disasters for individuals with chronic conditions was described as complex and challenging [49, 61–63, 70, 72, 79, 101, 105, 108, 117, 118, 128, 140], often requiring specialized transportation and equipment for mobility-impaired individuals [61, 63, 117]. Recognizing these risks, the Japanese government classifies individuals with chronic diseases as a priority group for evacuation assistance [108]. In Okinawa Prefecture, tsunami evacuation plans were tailored to their specific needs, including those requiring home oxygen therapy [118]. One study emphasized the need for collaboration among local governments, healthcare providers, and other stakeholders to address transportation, staffing, and medical supply shortages during evacuations of these individuals [70]. A Florida study found that while 7.1% of respondents needed special needs shelters, only 1.2% applied [101]. Evacuation decisions for individuals with chronic conditions were influenced by financial limitations, as those with lower incomes struggled to afford evacuation [49]. Strong family support networks also impacted decisions–among surveyed medically fragile US households, only 6.4% with strong family support evacuated, compared to 12.2% without such networks [49]. For Alzheimer’s Disease and Related Dementias patients, 85.2% of caregivers reported worsening symptoms during evacuation and feeling hesitant about evacuating, fearing shelters could not meet their needs and to face stigma [72].

##### Caregivers

Caregivers were identified as a vulnerable group, often residing in flood-prone areas due to economic constraints, which also limited their ability to adequately prepare for and respond to flood-induced disasters [111]. A study in rural Australia found that caregivers were nearly twice as likely to experience home flooding compared to other community members [111]. One study focused on Alzheimer’s Disease and Related Dementias caregivers in the US reported that, despite prior flood and hurricane experiences, many still felt unprepared to manage both caregiving and flood-induced disaster response [72]. Evacuation decision-making was described as particularly challenging: Alzheimer’s Disease and Related Dementias caregivers struggled with disrupting routines and familiar environments, essential for patient well-being [72], while nursing home caregivers had to balance the physical and emotional needs of residents with time constraints and logistical difficulties [117]. Concerns about privacy and stigma further complicated evacuation, with Alzheimer’s Disease and Related Dementias caregivers fearing judgement and lack of understanding from shelter workers [72]. Family, friends, and neighbors were reported to play a critical role by providing transportation, shelter, and support during flood-induced disasters [72, 95]. Access to community support programs was identified as a crucial relief measure for both caregivers and Alzheimer’s Disease and Related Dementias individuals [72].

#### Socioeconomic status

##### Economically unstable individuals

Studies used various terms to define economic vulnerability during flood-induced disasters, including low income [49, 53, 61, 66, 82, 84, 85, 91, 102, 118, 125–128, 132, 133, 139], poverty status [51, 61, 68, 74, 96, 103, 114, 131–133, 136], and a lack of material and/or financial resources [51, 59, 61, 95, 101, 111, 135]. Unemployment [91, 131–133, 136, 139] and occupation were also cited [84, 85, 91, 114, 136]. Economically unstable individuals were reported to face greater flood-induced disaster exposure, damage, and lower resilience [66, 102] during floods [85, 128, 131] and hurricanes [103]. A damage assessment of 1500 single-family homes post-Hurricane Ike revealed that poor communities of color were disproportionately affected [103]. Lower-income individuals were found to be less likely to receive and respond to disaster warnings compared to higher-income individuals [68]. Patterns of economic vulnerability were observed to cluster in urban areas in Rotterdam and Dordrecht [53], where economically unstable groups were more likely to inhabit substandard housing [102] and have the least resources for recovery from flood-induced disasters than more economically stable groups [111]. Systemic inequalities, including redlining and environmental racism, further trapped these communities in high-risk flood [74, 111] and hurricane [103] zones with poor infrastructure [135]. Occupation significantly influenced vulnerability: workers in fishing, agriculture, and mining faced job displacement and limited access to essential services after flood-induced disasters [87, 132]. In Zeeland, Netherlands, self-employed individuals and those employed in the hospitality sector were identified as at higher risk during flood-induced disasters [85]. Economically unstable individuals suffered greater health risks during flood-induced disasters [102, 135]. Following Hurricane Harvey, flooding disrupted transportation infrastructure in Texas, restricting evacuation and medical care access for these individuals [126]. A contrasting finding in England reported that households with multiple deprivations were less affected by floods compared to other households, likely because they were concentrated in urban areas with better emergency access [97]. Flood-induced disasters also caused long-term displacement and homelessness. After Hurricanes Katrina and Rita, 372,000 students from low-income families were displaced and experienced prolonged educational disruptions [125]. Poverty was found to strongly predict property damage in the US, where counties with higher poverty rates had greater flood-related losses (*p* < 0.01) [96]. Reduced access to disaster relief [118], food, water, healthcare, and other essentials significantly hindered recovery for low-income households [91]. In Quebec, Canada, individuals earning less than CAD 20,000 per year had lower post-flood adaptation rates, with only 25,63% taking adaptive measures following floods [82]. Low-income individuals and manufacturing workers were less likely to evacuate compared to more financially stable individuals, due to financial constraints, fear of job loss, and discrimination concerns [66, 88, 114]. During Hurricane Harvey, economic and transportation barriers forced low-income populations to remain in affected areas [126]. In South Florida, financially unstable residents were more likely to need evacuation assistance compared to more financially stable individuals, but less likely to respond to official warnings and evacuate than more economically stable individuals [61]. A study on Hurricane Florence’s post-disaster needs found that although lower-income individuals suffered more damage than higher-income ones due to substandard housing, median household income did not predict shelter needs, possibly because many lacked resources to access shelters [102]. An evacuation analysis during Hurricane Dorian showed that socioeconomically disadvantaged communities were less likely to evacuate compared to other communities (β = −0.25, *p* < 0.01) and, when they did, they stayed closer to their original locations (β = −0.54, *p* < 0.01) [115]. Only 76,7% of medically vulnerable, low-income households could sustain themselves financially for five days during an evacuation, compared to 86,3% of higher-income, non-medically fragile households [49]. However, in the US, during COVID-19, a higher evacuation rate was reported among low-income respondents [128] and public shelters in Florida were primarily used by those with the fewest economic resources [101].

##### Individuals with lower levels of education

Studies highlighted the increased vulnerability of individuals with primary or secondary education during flood-induced disasters [63, 82–84, 91, 95, 101–103, 121, 127, 128, 131–133, 136, 137, 141, 142]. A lack of higher education and limited disaster risk awareness were found to reduce the ability to understand and implement risk management strategies [91, 132]. In Canada, individuals without a high school diploma had lower flood adaptation rates (32.94%) compared to those with graduate degrees (57.32%) [82].

#### Social and cultural marginalization

##### Ethnic minorities

Various ethnic minorities were identified, such as Black [96, 97, 103, 118, 132], African-American [102, 103, 133, 135, 136], Hispanic/Latino [66, 96, 132, 133, 136], Asian [76, 96, 97, 102, 133, 135], and Indigenous populations [84, 91, 107, 137], including Native Americans [96]. Ethnic minorities were reported to face disproportionate risks during flood-induced disasters, with compounded social vulnerability [51, 66, 68, 86, 102, 132] and increased exposure to floods [73, 102, 103, 105, 132]. A US-based study identified Asian population as a highly at-risk group for floods and shelter access barriers [135]. Minorities were found to be more likely to reside in flood-prone areas compared to other groups [128, 133]. In New York, higher socio-vulnerability and climate displacement scores were correlated with a higher proportion of Black and Hispanic/Latino residents [132]. Further evidence from New York revealed that, under moderate coastal flooding, 27,000 African Americans, 8000 Hispanics, and 5000 Asians lived in NYC areas with high flood risk and low adaptive capacity [133]. Hurricane Ike’s aftermath highlighted increased effects on low-income communities of color [103], while another US study found higher property damage ratios in counties with higher Native American, Black, and Hispanic populations [96]. Disproportionate tsunami-related fatalities among Asian communities in the US were linked to mobility constraints, language barriers, and lack of hazard awareness [102, 135]. After Hurricane Katrina, Black mortality rates were reported to be four times higher than White populations, especially among elderly Black individuals, a disparity linked to economic disadvantage, residential segregation, and evacuation challenges [96]. Hispanic/Latino populations in New Jersey self-reported higher levels of medical concerns and limited healthcare access during Hurricane Sandy (3.4 ± 0.1 out of 5) [66]. Ethnic minorities were found to face barriers to disaster response and recovery due to limited resources and lower adaptive capacity [73, 102]. They were also less likely to receive and respond to warnings during flood-induced disasters compared to non-minority individuals [68]. Concerns over law enforcement interactions deterred some minorities from using public emergency services [105]. A study on post-Hurricane Florence needs found that for each 1% increase in minority population, per capita reported emergency needs decreased by 0.008, and food needs by 0.005 (B = − 0.008, *p* = 0.001; B = − 0.005, *p* = 0.013) [102]. However, in the UK, ethnic minorities had greater access to ambulance service during floods, possibly due to higher urban residency rates [97]. Financial constraints were found to limit evacuation options [114]. Additionally, discrimination in public shelters and host communities was reported to deter evacuation: during Hurricane Katrina, minority evacuees faced systemic and institutional racism [96], while Indigenous evacuees reported discrimination, with some concealing their backgrounds to avoid prejudice [107]. Indigenous communities were also described as facing fears of social services intervention, loss of traditional livelihoods, identity crises, and emotional and spiritual distress [107]. Evidence from Hurricane Katrina, Hurricane Florence, and studies involving Indigenous communities highlighted how the lack of culturally appropriate emergency communication exacerbated racial disparities in preparedness, information access, and evacuation response, limiting minorities’ ability to understand orders and access support [91, 102]. In Saudi Arabia, conservative Muslim communities faced evacuation challenges during floods due to cultural traditions [95]. Distrust in the government and fear of deportation discouraged undocumented individuals from evacuating during Hurricane Florence [102]. These barriers contributed to lower evacuation rates among ethnic minorities, leaving them at greater risk during flood-induced disasters [91, 96].

##### Migrants

Language and cultural barriers were reported to pose significant challenges for migrants during flood-induced disasters: in Rotterdam, Netherlands, migrants unfamiliar with the local language and emergency procedures struggled with flood preparedness and evacuation [53]; in Chile, they faced heightened tsunami vulnerability due to territorial unfamiliarity, language barriers, and limited awareness of emergency plans and disaster response protocols [91]; in Nebraska, US, language barriers caused frustration to flood-affected migrants [112]. Fear of government intervention and legal repercussions further discouraged undocumented migrants from seeking emergency services: studies in the US reported that they avoided public emergency services following hurricanes [102, 105] and floods [112], fearing deportation, negative impacts on legal status, or compromising their path to citizenship. Marginalized living conditions were found to compound migrant vulnerability: in Jeddah, Saudi Arabia, informal settlements with a high concentration of undocumented migrants suffered disproportionate impacts from flash floods, experiencing prolonged displacement and lacking access to relief services [95].

##### Individuals facing linguistic barriers

Studies highlighted that limited proficiency in the dominant language increased vulnerability during flood-induced disasters, restricting access to early warnings, critical resources, and disaster response service, contributing to social isolation, and preventing individuals from understanding safety instructions [17, 56, 61, 80, 91, 102, 112, 131, 133, 136, 141]. In New York, individuals with limited English proficiency were found to be less likely to be aware of flood warnings or follow safety measures [133]. Following Hurricane Florence, the proportion of individuals over age five who did not speak English was identified as a negative predictor of per capita reported emergency, food, and shelter needs (B = − 0.038, *p* < 0.001; B = − 0.026, *p* < 0.001; B = − 0.011, *p* < 0.001), likely due to limited access to translators or aid request guidance [102]. During the 2019 Nebraska floods, the disaster response failed to anticipate language-related needs, prompting local organizations to step in and bridge communication gaps [112]. In Chile, migrants unfamiliar with the local language struggled to understand emergency plans and recovery procedures during tsunamis [91]. Survey data from European disaster management experts ranked non-native speakers as among the most vulnerable to floods [131].

##### Tourists

A smaller number of studies examined tourists’ heightened vulnerability during flood-induced disasters, due to their temporary presence in unfamiliar locations [69, 78, 81, 88]. A study in Oregon highlighted a significant tourist population in tsunami-prone areas, raising concerns about shelter locations and overall safety measures for non-residents [81]. Research on tsunami evacuation planning in Canada emphasized that tourists were often concentrated in high-exposure areas and were less prepared for flood-induced disasters than the local population, due to their lack of knowledge of emergency protocols or experience with evacuation drills [78]. During Hurricane Irma in Florida, visitors in beachfront hotels faced heightened risks from storm surges and flooding [88]. Following the 2004 Sanjou floods, Japan recognized that individuals lacking regional knowledge struggled to identify safe evacuation areas, which led to their inclusion in the People Requiring Assistance During a Disaster classification [69].

##### LGBTIQ+

Flood-induced disasters were reported to exacerbate discrimination and marginalization, exposing LGBTIQ+ individuals to religious stigmatization, loss of safe spaces, and heightened risks of harassment and violence [71]. Emergency shelters and services often perpetuated exclusion through heteronormative policies, creating barriers for same-sex families and LGBTIQ+ individuals [71]. Trans individuals experienced even greater marginalization, with 44.4% reporting harassment–including verbal abuse, physical violence, intimidation, and denial of services–compared to 34.6% of LGB respondents in Australia [71]. Evidence indicated that LGBTIQ+ individuals were less likely to evacuate compared to non-LGBTIQ+ populations, due to pre-existing social vulnerabilities [115], and to prior experiences of discrimination, which contributed to feelings of insecurity and detachment from their communities [71]. Displacement further disrupted access to gender-affirming resources (e.g., clothing) that were critical to gender identity and self-expression [71]. Many relied on existing queer and trans community networks for support, using social capital to navigate discriminatory response systems [50].

##### Individuals experiencing social isolation

Social isolation was reported to increase flood vulnerability by limiting access to critical evacuation networks and support systems [49, 88, 114]. Weaker social connections were linked to delayed evacuations and increased flood exposure in Japan and the US [114], as well as during Hurricane Katrina, where individuals with fewer social ties were found to be less likely to evacuate compared to individuals with stronger social connections [88]. However, a study on medically fragile populations during Hurricane Irene found that strong family networks reduced evacuation likelihood: for those with strong family support, the odds of evacuation decreased by 0.611, whereas those without such networks were twice as likely to evacuate [49]. Knowing a neighbor’s name had no measurable impact on evacuation decisions for non-medically fragile individuals but was shown to increase evacuation likelihood for the medically fragile, suggesting that neighbors may have encouraged them to evacuate [49].

#### Living conditions, location, and transportation vulnerabilities

##### Individuals living in unstable housing or informal settlements

Economically [53, 61, 85, 102] and socially disadvantaged populations, including minorities [96], were reported to be more likely to reside in unstable or inadequate housing compared to other groups, exacerbating their exposure to floods and hurricanes [73]. Housing density and overcrowding were found to complicate coping with flood-induced disasters [133, 136], evacuations [133], and response and recovery efforts [91]. Structurally inadequate housing, such as mobile homes, was shown to be highly susceptible to floods and hurricanes, often sustaining severe damage or destruction [61, 88, 131, 133, 136]. In South Florida, many mobile home residents subject to mandatory evacuation orders were reported to be unable or unwilling to evacuate due to financial constraints [61]. A study found that 54% of disaster experts identified mobile home residency as a major flood vulnerability factor [131]. In Michigan, residents of structurally unsound homes expressed fear that they would not be able to evacuate safely [128]. Unreinforced masonry buildings were observed to be particularly prone to flood damage [96], and homes with fragile walls or precarious floors were found to be at greater risk of tsunami-related structural damage [91]. In La Guérinière, France, 60% of houses were reported to be single-story structures without flood adaptation, increasing resident risk [87]. In Florida, low-lying facilities were identified as highly vulnerable to hurricanes, leading to pre-arranged evacuation agreements with facilities on higher ground [103]. In Japan, individuals with special needs were found to have failed to use upper floors for evacuation, due to storage clutter, lack of handrails, or absence of emergency supplies, further increasing their flood vulnerability [94]. In the Netherlands, the construction year of buildings was used as a proxy for structural vulnerability, with older buildings more structurally vulnerable to floods compared to newer ones [53]. A study in New York found that high-rise, high-density public housing posed unique and underexplored risks during flood-induced disasters [68]. Lower median home values were linked to higher emergency (B = −1.527E-8, *p* = 0.006) and food needs (B = −0.768, *p* = 0.017) after hurricanes in the US [102]. A decline in flood-insured homeowners corresponded to increased emergency needs (B = − 1.019, *p* < 0.001) after hurricanes in the US [102], and lack of insurance was linked to greater property damage post-flood [96]. Renters were described as facing significant flood vulnerabilities [136, 138], including forced displacement, financial instability [74] and lack of control over property resilience [53]. Post-flood, landlords were reported to have forced tenants to vacate damaged properties, pushing them into a scarce and expensive rental market [74, 138]. In Rotterdam, Netherlands, renters were more likely to live in socially vulnerable neighborhoods, with rental occupancy averaging 75% compared to 25% for private ownership, and to have little incentive to invest in flood preparedness, as they may relocate [53]. Studies in the US and the Netherlands found that rental-heavy areas experience higher flood-related property damage [85, 96]. In Zeeland province, Netherlands, low-income renters faced compound economic vulnerabilities, including lower housing values, higher proportions of single-person households, and limited vehicle ownership [85]. Homeless individuals were identified as among the most vulnerable to floods, tsunamis, and hurricanes [57, 84, 91, 99, 103, 125, 131]. Homelessness was identified as a major contributor to flood-induced disaster vulnerability by disaster management experts [131]. Homeless individuals were found to be disproportionately exposed to hazardous environments, extreme weather, and lack of sanitation and clean water, compounding their vulnerability [91]. In New York, Hurricane Sandy was reported to have increased homelessness and deteriorated chronic health conditions and access to care among those already homeless [57]. Homeless children were described as facing educational disruptions, physical and psychological distress, and prolonged displacement, with flood-induced disaster identified as a primary driver of student homelessness in the US [125]. In Chile, additional post-tsunami aid was required from individuals living in unstable housing for basic survival, hygiene, and medical care [91]. A study on Roma communities in Romania found that recurring floods destroyed makeshift homes, left residents without shelter, spoiled food supplies, and rendered clothing unusable [99].

##### Individuals living in geographically vulnerable and isolated areas

Geographic location was identified as a key predictor of flood-related property damage, with individuals in flood-prone areas experiencing greater destruction and economic losses in both the US and Spain [96, 134]. Coastal populations were found to face heightened risks due to physical flood exposure and underlying socioeconomic vulnerabilities, such as poverty and inadequate infrastructure [17, 59, 86]. Urban environments were described as increasingly susceptible to flood-induced disasters due to unplanned land use, infrastructure deficiencies, environmental degradation, marginalization of vulnerable groups, and population density [91, 141, 143]. In Chile, dense urban communities were reported to exhibit lower resilience to tsunamis compared to open spaces [79, 91]. Studies on flood risk in the Netherlands [85] and storm surge vulnerability in coastal Virginia [59] confirmed that densely populated urban areas faced greater flood risk and experienced more severe consequences from flooding, including greater property loss potential. Limited access to shelters was identified as a key challenge, particularly for low-income urban residents who relied on public transportation in flood-prone neighborhoods [135]. Rapidly expanding cities with poor road connectivity [96] and peri-urban areas [134] were also found to be more vulnerable to flooding than areas with different characteristics. However, urban areas were observed to have better access to hospitals, emergency medical services, and shelters, making residents less isolated than those in rural areas [122]. Rural populations were identified as being at higher flood risk, with 49% of surveyed disaster management professionals ranking rural residency as a major vulnerability factor [131]. In the US, living in rural areas was identified as the strongest predictor of flood-related fatalities [96]. Rural communities were described as lacking essential resources and infrastructure, including fewer hospitals, shelters, and ambulance services [59, 97]. Property damage and financial losses were reported to be greater in US rural counties due to weaker flood-protection infrastructure and fewer resources for mitigation [96]. Many small rural communities were found to struggle with disaster response alone, as relief efforts often faced access challenges [112]. In flood-prone regions, isolated communities frequently lacked shelters, schools, and nursing homes, making disaster response and recovery difficult [134]. In Japan, residents in mountainous regions were reported to have experienced greater difficulty evacuating due to geographical constraints and long distances to shelters, which delayed response times and increased risk [93]. Similarly, South Korean mountain communities prone to isolation were described as being frequently cut off by flooding, requiring pre-identified evacuation routes and emergency plans to mitigate risk [119].

##### Individuals without access to personal transportation

Lack of private transportation was identified as a major evacuation barrier, forcing individuals to rely on walking, cycling, or inefficient public transit [133]. During Hurricane Sandy in New Jersey, Hispanics were found to be more likely to walk to evacuation centers than Blacks or Whites, indicating lower car ownership among this group [66]. Those without vehicles were described as being highly dependent on public transportation or emergency transit services, which often became unreliable or unavailable during flood-induced disasters [65, 85, 102, 105, 135], increasing their risk of being stranded in high-risk areas and unable to evacuate [98] and reach medical care and essential equipment [105]. In the US, the distance to the nearest emergency evacuation pickup point was identified as a key factor in evacuation vulnerability, since individuals unable to leave independently and not pre-registered for assistance, were often found to depend on neighbors for transport, resulting uncertainty and delays [65]. After Hurricane Andrew, South Florida’s already limited public transit system collapsed, leaving individuals without cars stranded, forced to walk to shelters, or unable to evacuate at all [61]. A study on US flood shelter access found that even in areas with high shelter availability, individuals without vehicles faced major barriers to reaching them [135]. In Zeeland, Netherlands, only 60% of residents were reported to own personal vehicles, making rail transport a crucial component of emergency mobility during floods [85].

#### Intersectionality during flood-induced disasters

**Fig. 4:**
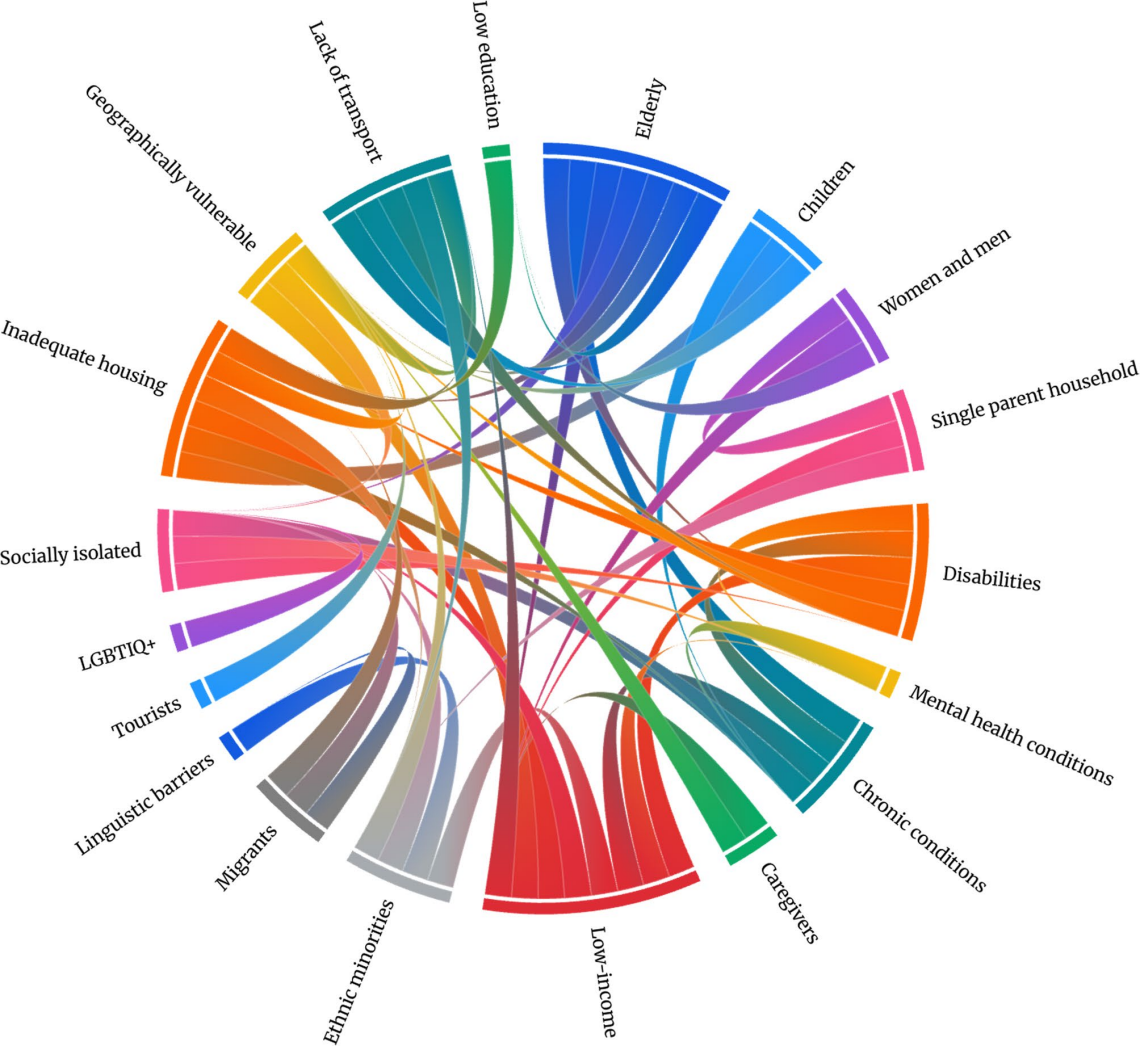
Intersectionality among identified vulnerable groups during flood-induced disasters

The collected evidence highlighted overlapping vulnerabilities among multiple groups during flood-induced disasters. Figure 4 provides a visual synthesis of these intersections, showing that certain groups–such as individuals with low income, those in inadequate housing, persons with disabilities or chronic conditions, the elderly, and socially isolated individuals–were frequently reported in the literature as intersecting with multiple other vulnerability factors. By contrast, categories such as tourists, LGBTIQ+ individuals, those facing linguistic barriers, individuals with mental health conditions, and those with lower levels of education appeared less frequently in intersectional profiles, suggesting areas of limited exploration in the literature.

### Key aspects in flood-induced disaster evacuation of vulnerable individuals

Several studies either proposed or implemented strategies to support the identification and evacuation of vulnerable individuals during flood-induced disasters or highlighted existing gaps and the need for such approaches. The evidence gathered is summarized in Table 5.

**Table 5:** Identification strategies of vulnerable individuals during flood-induced disasters

Country	Strategies for the identification of vulnerable individuals	References
Japan	Vulnerability registries to support evacuation efforts during floods and tsunamis	[60, 69, 93, 108, 110, 118]
	Annually updated “vulnerable people list” by the Shimobara Community Disaster Prevention Organization	[110]
	Kanazawa and Kochi Disaster Preparedness System (K-DiPS) to document medical conditions and needs of children with neurodevelopmental disorders during tsunamis	[108]
	GIS-based mapping and demographic profiling in Mabi to assess shelter capacity, accessibility and vulnerable population distribution	[93]
	GIS-based mapping and demographic profiling in Okinawa Prefecture to map COPD patients’ homes in relation to tsunami evacuation shelters	[118]
	Estimation of evacuation demand for large-scale flood in Tokyo.	[60]
	A survey in Minamisoma City municipality (Fukushima) identified individuals with disabilities requiring evacuation assistance and ongoing support	[69]
United States	Identifying the location of socially vulnerable communities enables planners to better allocate resources and support community-based disaster preparedness efforts	[103]
	Analysis of the intersection of social vulnerability and sea level rise using cluster analysis on 13 social indicators and flood and population exposure modeling in Miami-Dade County	[127]
	In Panama City, Florida, development of a GIS-based approach to improve special needs shelters accessibility and repurpose regular shelters	[130]
	Estimation of the population at risk of flooding combined with their level of vulnerability using GIS in Texas and West Virginia	[136]
	Development of a demand model to simulate household evacuation behaviors under compound hazard scenarios	[142]
United Kingdom	Assessment of ambulance-assisted evacuations of care home residents during flooding in Norfolk and Suffolk	[90]
	Hotspot analysis to identify vulnerable facilities where emergency coverage was insufficient	[97]
Spain	Development of flood risk maps identifying dependent populations, shelter deficits, isolation levels, building vulnerability, and critical sites in Málaga	[134]
Chile	Simulation of a worst-case tsunami scenario in Cartagena Bay to evaluate social vulnerability using the Social Vulnerability Index (SoVI) and census data	[91]
Australia	Service providers highlighted concerns about the lack of centralized records for vulnerable populations and of comprehensive databases to track individuals requiring specialized care	[62]
Canada	GIS mapping tools for flood risk assessment and evacuation planning of socially vulnerable populations	[54]
South Korea	Automated analysis tool to identify in real time at-risk communities, isolated areas, roads, buildings, population density, evacuation routes and nearby shelters in mountainous areas	[119]
Romania	GIS modelling to analyze suitability and accessibility of evacuation points, based on historical flood data in rural mountainous areas	[113]
Italy	Use of registries to identify individuals with disabilities, the elderly, those under dialysis, ventilation, and home care, and of just-in-time phone calls to identify unknown vulnerable individuals	[17]

The included studies highlighted various sheltering options during flood-induced disasters, including medical and non-medical ACFs, vertical evacuation, and sheltering-in-place. Evidence is summarized in Table 6.

**Table 6:** Sheltering options during flood-induced disasters identified in the included studies

Sheltering options	References
**Alternative Care Facilities**	
***Medical ACFs***	
Special Needs Shelters, Special Medical Needs Shelters, Emergency Shelters for Vulnerable Populations	[57, 104, 108, 130]
Unaffected healthcare facilities	[17, 48, 135]
Elderly care facilities	[17]
Mental health facilities	[17]
High-dependency rehabilitation facilities	[17]
Nursing homes	[17]
Day centers	[17]
***Non-medical ACFs***	
Temporary evacuation centers	[69, 128]
Smaller shelters for vulnerable individuals	[109]
Community facilities in general	[69, 75, 98, 128, 135]
Hotels, dormitories, and motels (particularly during the concurrent pandemic, and for Alzheimer’s patients)	[17, 103, 128]
Schools	[17, 52, 60, 64, 69, 98, 113, 123, 128, 135]
Sports facilities, gymnasiums, and sports halls	[17, 64, 75, 113, 123]
Religious centers and churches	[128, 135]
Theatres	[113]
Disused factories	[75]
Friends or family members’ homes and other personal residences	[128]
**Other sheltering options**	
Vertical evacuation (i.e., seeking refuge in multi-story buildings or elevated structures)	[79, 81, 106, 140]
Sheltering in place (particularly for those with limited mobility)	[69]

## Discussion

This scoping review examined how vulnerability is conceptualized, addressed, and operationalized in the context of evacuations during flood-induced disasters in HICs. It mapped the existing literature on vulnerable population groups and related identification and evacuation strategies, revealing several persistent limitations.

The coverage of vulnerability in flood-induced disaster evacuations remains incomplete. Populations such as single-parent or non-nuclear households, pet owners, pregnant women, individuals with mental health conditions, caregivers, those with lower educational attainment, tourists, socially isolated individuals, and those without private transport are underrepresented. Other potentially vulnerable groups–such as prisoners, international students, seasonal or undocumented workers, informal caregivers, emergency responders, religious minorities, and victims of domestic abuse–are absent from the literature, underscoring a pressing need for greater inclusivity in research and practice.

The analysis of intersectionality across included studies revealed that certain groups were rarely represented, highlighting persistent challenges in capturing the complex and overlapping dimensions of vulnerability. The findings of this review largely reproduce the group-based and factor-specific approaches that dominate the existing literature, as evident from Tables 3 and 4. The limitations of such approaches are well recognized in recent scholarship, which frames vulnerability as layered and dynamic rather than categorical or static [35, 146]. Emerging perspectives further advocate “challenges-based” models that prioritize functional needs–such as communication, mobility, safety, and access to care–over fixed population categories [34]. These shortcomings become particularly evident when considering that, at the individual level, vulnerability emerges from a unique constellation of circumstances, making vulnerability-informed evacuation management inherently complex. Not all individuals within a defined vulnerable group experience the same degree or type of risk. For instance, a person with a chronic illness but stable resources may face fewer challenges during a flood-induced disaster than someone who becomes vulnerable due to temporary circumstances, such as an injury or lack of transport.

Despite these conceptual and methodological constraints, the findings provide valuable signals and group identifiers. The categories should be regarded not as fixed or uniform, but as analytical entry points to understand the diversity of needs within affected populations. Used in this way, the findings can support disaster managers in moving beyond group labels and in operationalizing intersectionality when identifying and assisting at-risk subpopulations. The combination of vulnerabilities is what ultimately matters, varying across exposure and intervention contexts and producing distinct configurations of risk within affected populations. Vulnerability should be understood as a fluid, multilayered condition that evolves over time rather than a fixed state. As described in the concept of the vulnerability vortex [147], biological, social, and environmental factors interact dynamically, amplifying or mitigating one another and shaping individuals’ ability to respond and recover. This reinforces the view that vulnerability is relational, not additive, and calls for disaster management strategies capable of addressing both singular and compounding vulnerabilities throughout the evacuation process.

A hybrid approach that combines the anticipatory value of group-based identification with the flexibility to detect emergent, situational vulnerabilities offers the most promising pathway toward inclusive and context-sensitive evacuation management. Translating this into practice requires dedicated systems, skilled personnel and interoperable tools for vulnerability identification and data sharing. In HICs, this can take the form of structured networks and trained personnel responsible for coordinating vulnerability management. In lower-resource settings, where capacities differ, community-based actors can serve as the backbone of vulnerability management, integrating local knowledge, social networks, and trust into disaster management.

The findings also highlight the limited availability of clear, actionable guidance for implementing vulnerability-informed evacuation strategies. Few studies explicitly link vulnerability assessment to operational outcomes, revealing a gap between theoretical frameworks and practical disaster management. Addressing this gap is critical to strengthening the evidence base for disaster risk reduction and underscores the contribution of this review in bridging vulnerability analysis with evacuation management in the context of flood-induced disasters. More specifically, there is limited evidence on how to identify vulnerable individuals and match them to the most appropriate sheltering options. While several studies introduce promising tools or protocols to support these processes, details regarding their scalability and integration into national disaster management systems are often absent or insufficiently documented. Moreover, although recent evidence highlights the potential of FSC implementation [148, 149], the contribution of ACFs to vulnerability-informed, needs-responsive evacuation remains underexplored. FSC is particularly relevant for vulnerable populations, who face heightened risks and often lack resources during disasters. It enhances community self-efficacy by training local individuals–such as elderly care staff or disability advocates–to serve as first responders and provide tailored support. This includes providing culturally sensitive training and establishing volunteer networks equipped to address diverse needs, from accessible evacuation to psychosocial aid. FSC also involves the strategic, decentralized pre-positioning of specialized supplies (e.g., specific medications, assistive technologies, or culturally appropriate food), as well as the adaptive use of non-traditional spaces for care and shelter that ensure privacy, accessibility, and integration with existing support networks. Further efforts should also focus on leveraging community-based resources as integral components of FSC. Non-medical ACFs such as community centers, fire stations, religious institutions, or sports facilities could serve as initial hubs for rapid vulnerability assessment and triage, directing individuals toward suitable sheltering and care options. Recognizing the multifaceted nature of community–as both a social network and a physical space–is essential, as each interpretation entails distinct operational implications for evacuation and vulnerability management. Realizing FSC potential depends heavily on inclusive planning and communication, consent-based vulnerability mapping, strong community participation, and equitable resource distribution–factors that remain difficult to operationalize in many settings.

Beyond this, it is essential to consider that vulnerability and evacuation management are not one-time or stand-alone processes but evolve across the disaster timeline. On one hand, vulnerability assessment supports preparedness and the early identification of at-risk groups at the onset of disasters; on the other, this process is closely intertwined with rapid needs assessment, which guides immediate response efforts and resource allocation. Integrating these two approaches within disaster management strategies would strengthen the continuity between evacuation planning and emergency service delivery, ensuring that both pre-existing vulnerabilities and newly emerging needs are systematically addressed. At the same time, vulnerability management does not end with the immediate response but extends into the recovery phase. Limited attention has been paid to the longer-term health and social effects of evacuation and relocation, which can themselves become sources of chronic vulnerability. Evidence shows that displacement and prolonged relocation are associated with sustained physical and mental health impacts–including chronic stress, disrupted continuity of care, and social isolation [150, 151]. These cascading effects are often most severe among vulnerable groups, perpetuating disadvantage and demonstrating that vulnerability can evolve into a long-term condition. Integrating post-evacuation recovery and long-term support into disaster management planning is therefore essential to prevent the recurrence or deepening of vulnerabilities over time.

These theoretical and operational gaps are particularly concerning given the increasing frequency and severity of flood-induced disasters across HICs. Recent events in Europe alone illustrate the growing urgency of developing vulnerability-informed evacuation strategies. Italy’s Emilia-Romagna region, for instance, has endured four major floods since 2023, including one that required the evacuation of over 36,600 people [17]. In 2024, Storm Boris triggered widespread flooding across Central and Eastern Europe, displacing tens of thousands in the Czech Republic, Austria, Poland, Romania, and Slovakia [152, 153]. The same year, Spain’s Valencia region suffered devastating floods that caused 216 fatalities–nearly half among elders over 70–and displaced more than 120,000 individuals [154, 155]. These events underscore the pressing need for vulnerability-informed evacuation strategies.

The findings of this review are relevant to researchers, policymakers, and professionals in disaster management, public health, and social care. The systematic tables and mapping can serve as practical tools for inclusive and comprehensive disaster management plans and protocol development. Although centered on flood-induced disasters in HICs, the insights may extend to other hazards and contexts, bearing in mind that different types of events and activities reveal distinct mechanisms of vulnerability: some hazards may amplify health-related risks, while others may highlight infrastructural or mobility constraints. Likewise, in low- and middle-income countries, weaker social protection systems and resource limitations can produce qualitatively different vulnerability configurations.

Limitations of the study include the predominance of studies from the United States and Japan, potentially affecting the generalizability of findings to other HICs. Additionally, inconsistencies in terminology, conceptual definitions, and methodological approaches across the included literature posed challenges to thematic comparison and restricting inclusion to English-language, peer-reviewed publications may have introduced both publication and language bias.

Future research should comparatively assess how vulnerability patterns differ across hazards, activities, and population contexts, and examine recent flood-induced disasters in HICs to document how vulnerability has been operationalized in evacuation planning and practice. Comparative case studies could provide empirical evidence on context-specific strategies and their effectiveness in protecting diverse populations. Participatory research approaches–such as community-based focus groups–may help refine vulnerability from the perspective of affected populations and strengthen exchange between disaster management researchers and professionals. Finally, testing and scaling digital tools, such as GIS-based platforms or mobile applications for real-time vulnerability and evacuation needs mapping represent a promising direction for innovation in vulnerability-informed disaster management.

## Conclusion

This scoping review identified critical evidence gaps in how vulnerability is integrated into evacuation management for flood-induced disasters in HICs.

The findings reveal that vulnerability remains incompletely represented in the literature, with several groups–such as single-parent households, caregivers, individuals with mental health conditions, and undocumented workers–largely overlooked. Categories such as tourists, LGBTIQ+ individuals, those facing linguistic barriers, individuals with mental health conditions, and those with lower levels of education appeared less frequently in intersectional profiles, indicating persistent blind spots and limited exploration of overlapping vulnerabilities. The reviewed literature also reveals considerable variation in how different countries identify and manage vulnerable populations. Japan stands out for its use of vulnerability registries, GIS-based mapping, and dedicated preparedness systems for specific groups such as pregnant women and persons with disabilities. Other contexts, including the United States, United Kingdom, Spain, and Italy, have implemented tools such as social vulnerability mapping, ambulance-assisted evacuation registries, and just-in-time identification protocols, though their coverage and integration remain uneven. Likewise, the literature documents wide variation in sheltering strategies for vulnerable populations–from medical ACFs (e.g., special needs shelters, care facilities) to non-medical ones such as schools, sports halls, and community centers–highlighting diverse but often fragmented models of inclusive evacuation management. The reviewed evidence also indicates that vulnerability is seldom linked to explicit evacuation management, limiting the translation of assessment findings into operational measures. Evidence on the application of FSC and the use of ACFS for vulnerability-informed evacuation remains scarce, particularly regarding the potential of non-medical facilities as community-based hubs for rapid vulnerability assessment and triage. Furthermore, the literature underscores the need to strengthen continuity across the temporal phases of disaster management to address pre-existing, cascading vulnerabilities and emerging needs.

Ultimately, this review underscores that vulnerability is not a fixed attribute, but an evolving and multilayered condition shaped by intersecting factors and contextual dynamics. The findings make clear that vulnerabilities are neither uniform within nor between groups but instead combine and interact in ways that differ across social, environmental, and operational contexts. This interdependence makes need-centered, vulnerability-informed evacuation management inherently complex, requiring flexible approaches that can adapt to diverse and changing realities. Rather than being used as static group lists, the identified categories should serve as practical tools for operationalizing intersectionality–helping policymakers and disaster management professionals translate nuanced understandings of vulnerability into inclusive, context-responsive evacuation strategies.

## Electronic supplementary material

Below is the link to the electronic supplementary material.

Supplementary Material 1

## Data Availability

All data analyzed are from published sources cited in the manuscript. The extraction dataset is available from the corresponding author upon reasonable request.

## Abbreviations

ACFs: Alternative Care Facilities
COPD: Chronic Obstructive Pulmonary Disease
ED: Emergency Department
EU: European Union
FSC: Flexible Surge Capacity
HICs: High-Income Countries
NCDs: Non-Communicable Diseases
PRISMA-ScR: Preferred Reporting Items for Systematic Reviews and Meta-Analyses extension for Scoping Reviews
PTSD: Post-Traumatic Stress Disorder
SoVI: Social Vulnerability Index
SVEAI: Social Vulnerability for Evacuation Assistance Index

## References

[1] P. Hazard. https://www.preventionweb.net/understanding-disaster-risk/component-risk/hazard. (accessed 2025).

[2] P. Hazard information profiles (HIPs) - flood. https://www.preventionweb.net/hips-cluster/flood. (accessed 2025).

[3] Intergovernmental Panel on Climate Change (IPCC). Climate Change 2021 – The Physical Science Basis. Cambridge: Cambridge University Press; 2023.

[4] Romanello M, et al. The 2024 report of the Lancet countdown on health and climate change: facing record-breaking threats from delayed action. The Lancet. 2024;404(10465):1847–96. 10.1016/S0140-6736(24)01822-1. 10.1016/S0140-6736(24)01822-1 PMC7616816 39488222

[5] Jongman B, Ward PJ, Aerts JCJH. Global exposure to river and coastal flooding: long term trends and changes. Glob Environ Change. 2012;22(4):823–35. 10.1016/j.gloenvcha.2012.07.004.

[6] CRED. EM-DAT the International disaster database - Centre for Research on the epidemiology of disasters (CRED). https://public.emdat.be/ (accessed 2025).

[7] Ezzatvar Y, López-Gil JF. Urgent call for enhanced flood preparedness and response in Spain. Lancet. 2024;404(10470):2419–20. 10.1016/S0140-6736(24)02506-6. 10.1016/S0140-6736(24)02506-6 39615505

[8] Liu Q, et al. Global, regional and national trends and impacts of natural floods, 1990-2022. Bull World Health Organ. 2024, Jun, 1;102(6):410–20. 10.2471/BLT.23.290243. 38812801 10.2471/BLT.23.290243 PMC11132161

[9] Coppola D. Introduction to international disaster management. 4th. Elsevier; 2020.

[10] CRED. 2023 disasters in numbers: a significant year of disaster impact. 2023. [Online]. Available: https://www.preventionweb.net/publication/2023-disasters-numbers-significant-year-disaster-impact.

[11] Koenig KL, Schultz CH. Koenig and Schultz’s disaster Medicine - comprehensive principles and practices. 2nd. Cambridge University Press; 2016.

[12] Abebe YA, Pregnolato M, Jonkman SN. Flood impacts on healthcare facilities and disaster preparedness - a systematic review. Int J Disaster Risk Reduct. 2025;119. 10.1016/j.ijdrr.2025.105340.

[13] Runkle JD, Brock-Martin A, Karmaus W, Svendsen ER. Secondary surge capacity: a framework for understanding long-term access to primary care for medically vulnerable populations in disaster recovery. Am J Public Health. 2012, Dec;102(12):e24–32. 10.2105/AJPH.2012.301027. 23078479 10.2105/AJPH.2012.301027 PMC3519329

[14] World Health Organization. Floods in the who European region: health effects and their prevention. 15(39):55. [Online]. Available: https://reliefweb.int/report/world/floods-who-european-region-health-effects-and-their-prevention. 05/20/2013. Accessed: 2024/01/29/.

[15] World Health Organization Regional Office for Europe. Floods and health - fact sheets for health professionals. 2014. [Online]. Available: https://iris.who.int/bitstream/handle/10665/375390/WHO-EURO-2014-8519-48291-71705-eng.pdf?sequence=1.

[16] Ahern M, Kovats RS, Wilkinson P, Few R, Matthies F. Global health impacts of floods: epidemiologic evidence. Epidemiol Rev. 2005;27:36–46. 10.1093/epirev/mxi004. 15958425 10.1093/epirev/mxi004

[17] Valente M, et al. The 2023 floods in the Emilia-Romagna region, Italy: A retrospective qualitative investigation into response strategies and criticalities. Int J Disaster Risk Reduct. 2025;116. 10.1016/j.ijdrr.2024.105089.

[18] European Union. Eu preparedness Union strategy to prevent and react to emerging threats and crises. 2025. [Online]. Available: https://civil-protection-humanitarian-aid.ec.europa.eu/news-stories/news/eu-preparedness-union-strategy-prevent-and-react-emerging-threats-and-crises-2025-03-26_en.

[19] United Nations Office for Disaster Risk Reduction (UNDRR). The sendai framework terminology on disaster risk Reduction “Vulnerability”. https://www.undrr.org/terminology/vulnerability. (accessed 2025).

[20] Birkmann J, et al. Framing vulnerability, risk and societal responses: the move framework. Nat Hazards. 2013;67(2):193–211. 10.1007/s11069-013-0558-5.

[21] Chan SW, Abid SK, Sulaiman N, Nazir U, Azam K. A systematic review of the flood vulnerability using geographic information system. Heliyon. 2022, Mar;8(3):e09075. 10.1016/j.heliyon.2022.e09075. 35284686 10.1016/j.heliyon.2022.e09075 PMC8914095

[22] Flanagan BE, Gregory EW, Hallisey EJ, Heitgerd JL, Lewis B. “A social vulnerability Index for disaster management,” (in en). J Homel Secur Emerg Manag. 8(1). 10.2202/1547-7355.1792. 01/5/2011.

[23] Cutter SL, Boruff BJ, Shirley WL. Social vulnerability to environmental Hazards*. Soc Sci Q. 2003;84(2):242–61. 10.1111/1540-6237.8402002.

[24] Jamshed A, Rana IA, Birkmann J, McMillan JM, Kienberger S. A bibliometric and systematic review of the methods for the improvement of vulnerability assessment in Europe framework: a guide for the development of further multi-hazard holistic framework. Jamba. 2023;15(1):1486. 10.4102/jamba.v15i1.1486. 38223542 10.4102/jamba.v15i1.1486 PMC10784246

[25] Ran J, MacGillivray BH, Gong Y, Hales TC. The application of frameworks for measuring social vulnerability and resilience to geophysical hazards within developing countries: a systematic review and narrative synthesis. Sci Total Environ. 2020, Apr, 1;711:134486. 10.1016/j.scitotenv.2019.134486. 31818578 10.1016/j.scitotenv.2019.134486

[26] Brannen DE, Branum M, Pawani S, Miller S, Bowman J, Clare T. “Medical allocations to persons with special needs during a Bioterrorism event,” (in eng). Online J Public Health Inf. 2016, 2016;8(3):e200. 10.5210/ojphi.v8i3.6977. 10.5210/ojphi.v8i3.6977 PMC5302466 28210421

[27] Dries D, et al. Special populations: care of the critically ill and injured during pandemics and disasters: chest consensus statement. Chest. 2014, Oct;146(4 Suppl):e75S-86S. 10.1378/chest.14-0737. 25144661 10.1378/chest.14-0737

[28] Tomio J, Sato H. Emergency and disaster preparedness for chronically ill patients: a review of recommendations. Open Access Emerg Med. 2014;6:69–79. 10.2147/OAEM.S48532. 27147882 10.2147/OAEM.S48532 PMC4753992

[29] Behr JG, Diaz R. Disparate health implications stemming from the propensity of elderly and medically fragile populations to shelter in place during severe storm events. J Public Health Manag Pract. 2013, Sep-Oct;19( Suppl 2):S55–62. 10.1097/PHH.0b013e318297226a. 23903396 10.1097/PHH.0b013e318297226a

[30] Fernandez LS, Byard D, Lin CC, Benson S, Barbera JA. Frail elderly as disaster victims: emergency management strategies. Prehosp Disaster Med. 2002, Apr-Jun;17(2):67–74. 10.1017/s1049023x00000200. 12500729 10.1017/s1049023x00000200

[31] Ma C, Qirui C, Lv Y. “One community at a time”: promoting community resilience in the face of natural hazards and public health challenges. BMC Public Health. 2023, Dec, 14;23(1):2510. 10.1186/s12889-023-17458-x. 38097956 10.1186/s12889-023-17458-x PMC10722774

[32] Evans J. Mapping the vulnerability of older persons to disasters. Int J Older People Nurs. 2010, Mar;5(1):63–70. 10.1111/j.1748-3743.2009.00205.x. 20925759 10.1111/j.1748-3743.2009.00205.x

[33] Cho SY, Chang H. Recent research approaches to urban flood vulnerability, 2006-2016, (in en). Natural Hazards. 2017;88(1):633–49. 10.1007/s11069-017-2869-4.

[34] United Nations Office for Disaster Risk Reduction (UNDRR). Bridging the gap between vulnerable groups and vulnerable situations: towards an integrative perspective on vulnerability for disaster risk reduction. 2022. [Online]. Available: https://www.undrr.org/publication/bridging-gap-between-vulnerable-groups-and-vulnerable-situations-towards-integrative.

[35] Luna F. Elucidating the concept of vulnerability: layers not labels. Int J Feminist Approaches To Bioethics. 2009;2(1):121–39.

[36] Painter MA, Shah SH, Damestoit GC, Khalid F, Prudencio W, Chisty MA, Tormos-Aponte F, Wilhelmi O. A systematic scoping review of the social vulnerability index as applied to natural hazards. Nat Hazards. 2024 Jun;120(8):7265–356. 10.21203/rs.3.rs-2978301/v1.

[37] Jeleff M, et al. Vulnerability and one Health assessment approaches for infectious threats from a social science perspective: a systematic scoping review. (In Eng), Lancet Planet Health. 2022 Aug;6(8):e682–e693. 10.1016/S2542-5196(22)00097-3. 10.1016/S2542-5196(22)00097-3 35932788

[38] Mah JC, Penwarden JL, Pott H, Theou O, Andrew MK. Social vulnerability indices: a scoping review. BMC Public Health. 2023 Jun 28;23(1):1253. 10.1186/s12889-023-16097-6. 37380956 10.1186/s12889-023-16097-6 PMC10304642

[39] Spielman SE, et al. Evaluating social vulnerability indicators: criteria and their application to the social vulnerability index. Nat Hazards. 2020;100(1):417–36. 10.1007/s11069-019-03820-z.

[40] Kim K, Kang J-Y, Hwang C. Identifying indicators contributing to the social vulnerability Index via a scoping review. Land. 2025;14(2). 10.3390/land14020263.

[41] Alam MDJ, Habib MA. Vulnerability assessment during mass evacuation: integrated microsimulation-based evacuation modeling approach, (in en). Transp Res Rec. 2019;2673(10):225–38. 10.1177/0361198119848409.

[42] Phattharapornjaroen P, Carlström E, Khorram-Manesh A. Developing a conceptual framework for flexible surge capacity based on complexity and collaborative theoretical frameworks. Public Health. 2022;208:46–51. 10.1016/j.puhe.2022.04.012. 10.1016/j.puhe.2022.04.012 35687955

[43] Glantz V, Phattharapornjaroen P, Carlström E, Khorram-Manesh A. Regional flexible surge capacity-A flexible response system. Sustainability. 2020;12(15):5984.

[44] Khorram-Manesh A. Flexible surge capacity - public health, public education, and disaster management. Health Promot Perspectives. 2020;10(3):175–9. 10.34172/hpp.2020.30. 10.34172/hpp.2020.30 PMC7420172 32802753

[45] Tricco AC, et al. PRISMA extension for scoping reviews (PRISMA-ScR): checklist and explanation. Ann Intern Med. 2018 Oct 2;169(7):467–73. 10.7326/M18-0850. 30178033 10.7326/M18-0850

[46] Peters M, Godfrey C, McInerney P, Munn Z, Tricco A, Khalil H. Chapter, 11: scoping reviews. JBI Reviewer’s Man. 2019.

[47] The World Bank. World Bank country and lending groups. https://datahelpdesk.worldbank.org/knowledgebase/articles/906519-world-bank-country-and-lending-groups. (accessed 04, 2025).

[48] Espiritu M, et al. Evacuation of a neonatal intensive care unit in a disaster: lessons from Hurricane Sandy. Pediatrics. 2014 Dec;134(6):e1662–9. 10.1542/peds.2014-0936. 25384488 10.1542/peds.2014-0936

[49] Ng M, Behr J, Diaz R. Unraveling the evacuation behavior of the medically fragile population: findings from Hurricane Irene. Transp Res Part A: Policy Pract. 2014;64:122–34. 10.1016/j.tra.2014.03.015.

[50] Thomas M, Tsujimoto T. Vulnerability to flood risks in Japanese urban areas: crisis management and emergency response for efficient evacuation management. WIT Trans Ecol Environ. 2014;(184):61–71. 10.2495/FRIAR140061.

[51] Vink K, Takeuchi K, Kibler KM. A quantitative estimate of vulnerable people and evaluation of flood evacuation policy. J Disaster Res. 2014;9(5):887–900. 10.20965/jdr.2014.p0887.

[52] Kim K, Pant P, Yamashita E. Evacuation planning for plausible worst case inundation scenarios in Honolulu, Hawaii. J Emerg Manag. 2015 Mar-Apr;13(2):93–108. 10.5055/jem.2015.0223. 25902293 10.5055/jem.2015.0223

[53] Koks EE, Jongman B, Husby TG, Botzen WJW. Combining hazard, exposure and social vulnerability to provide lessons for flood risk management. Environ Sciamp Policy. 2015;47:42–52. 10.1016/j.envsci.2014.10.013.

[54] Mioc D, et al. Natural and man-made flood risk mapping and warning for socially vulnerable populations. Int J Saf Secur Eng. 2015;5(3):183–202. 10.2495/safe-v5-n3-183-202.

[55] Sun JE, Lee JS, Hong WH. A study on improvement plans for evacuation and information communication method through the awareness survey of flood damage. Int J Appl Eng Res. 2015.

[56] Challender J. Storm surge impact to subterranean areas by Hurricane Sandy, and lessons for Japan’s storm surge countermeasures. J Disaster Res. 2016;11(2):274–84. 10.20965/jdr.2016.p0274.

[57] Doran KM, et al. Emergency department visits for homelessness or inadequate housing in New York City before and after Hurricane Sandy. J Urban Health. 2016 Apr;93(2):331–44. 10.1007/s11524-016-0035-z. 26979519 10.1007/s11524-016-0035-z PMC4835349

[58] Farmer AK, DeYoung SE, Wachtendorf T. Pets and evacuation: an ongoing challenge in disasters. J Homel Secur Emerg Manag. 2016;13(4). 10.1515/jhsem-2016-0051.

[59] Liu H, Behr JG, Diaz R. Population vulnerability to storm surge flooding in coastal Virginia, USA. Integr Environ Assess Manag. 2016 Jul;12(3):500–09. 10.1002/ieam.1705. 26295749 10.1002/ieam.1705

[60] Nakamura H. Possibilities of neighborhood evacuation within a district in the event of a large-scale flood in a low-lying area: a case study of Shinden district in Tokyo. E3S Web of Conferences. 2016;7. 10.1051/e3sconf/20160719005.

[61] Prasad S. Assessing the need for evacuation assistance in the 100 year floodplain of South Florida. Appl Geogr. 2016;67:67–76. 10.1016/j.apgeog.2015.12.005.

[62] Ryan BJ, et al. Reducing disaster exacerbated non-communicable diseases through Public Health infrastructure resilience: perspectives of Australian disaster service providers. PLoS Curr. 2016 Dec 21;8. 10.1371/currents.dis.d142f36b6f5eeca806d95266b20fed1f. 10.1371/currents.dis.d142f36b6f5eeca806d95266b20fed1f PMC5308209 28239511

[63] Weller SC, Baer R, Prochaska J. Should I stay or should I go? Response to the Hurricane Ike evacuation order on the Texas Gulf Coast. Nat Hazards Rev. 2016;17(3). 10.1061/(asce)nh.1527-6996.0000217.

[64] Fekete A, Tzavella K, Baumhauer R. Spatial exposure aspects contributing to vulnerability and resilience assessments of urban critical infrastructure in a flood and blackout context. Nat Hazards. 2016;86(S1):151–76. 10.1007/s11069-016-2720-3.

[65] Bian R, Wilmot CG. Measuring the vulnerability of disadvantaged populations during hurricane evacuation. Nat Hazards. 2017;85:691–707.

[66] Burger J, Gochfeld M, Pittfield T, Jeitner C. Responses of a vulnerable hispanic population in New Jersey to Hurricane Sandy: access to care, medical needs, concerns, and ecological ratings. J Toxicol Environ Health A. 2017;80(6):315–25. 10.1080/15287394.2017.1297275. 28644717 10.1080/15287394.2017.1297275 PMC5531200

[67] Coles D, Yu D, Wilby RL, Green D, Herring Z. Beyond ‘flood hotspots’: modelling emergency service accessibility during flooding in York, UK. J Hydrol. 2017;546:419–36. 10.1016/j.jhydrol.2016.12.013.

[68] Tai TWC, Lee JY, Bame SI. Longitudinal patterns of unmet needs during Texas floods, May-June 2015. Disaster Prev Manag. 2017;26(5):611–28. 10.1108/dpm-02-2017-0015.

[69] Yamasaki E, Hayashi H. People who cannot move during a disaster - initiatives and examples in Japan disaster victim support. J Disaster Res. 2017;12(1):137–46. 10.20965/jdr.2017.p0137.

[70] Khorram-Manesh A, Yttermyr J, Sörensson J, Carlström E. The impact of disasters and major incidents on vulnerable groups: risk and medical assessment of Swedish patients with advanced care at home. Home Health Care Manag Pract. 2017;29(3):183–90. 10.1177/1084822317699156.

[71] Gorman-Murray A, McKinnon S, Dominey-Howes D, Nash CJ, Bolton R. Listening and learning: giving voice to trans experiences of disasters. Gend Place Culture. 2017;25(2):166–87. 10.1080/0966369x.2017.1334632.

[72] Gibson A, Walsh J, Brown LM. A perfect storm: challenges encountered by family caregivers of persons with Alzheimer’s disease during natural disasters. J Gerontol Soc Work. 2018 Oct;61(7):775–89. 10.1080/01634372.2018.1474158. 29781774 10.1080/01634372.2018.1474158

[73] Hernandez D, et al. Public housing on the periphery: vulnerable residents and depleted resilience reserves post-Hurricane Sandy. J Urban Health. 2018 Oct;95(5):703–15. 10.1007/s11524-018-0280-4. 30088128 10.1007/s11524-018-0280-4 PMC6181816

[74] Mort M, Walker M, Williams AL, Bingley A. Displacement: critical insights from flood-affected children. Health Place. 2018 Jul;52:148–54. 10.1016/j.healthplace.2018.05.006. 29890442 10.1016/j.healthplace.2018.05.006

[75] Wagemann E, Moris R. Transitional habitability: solutions for post-catastrophe in Chile. Int J Disaster Risk Reduct. 2018;31:514–25. 10.1016/j.ijdrr.2018.06.007.

[76] Wilson MT. Catastrophes and their classifications: revising New York City’s Hurricane evacuation maps after Irene and Sandy. J Homel Secur Emerg Manag. 2018;15(2). 10.1515/jhsem-2016-0008.

[77] Huang G. Enhancing dialogue between flood risk management and road engineering sectors for flood risk reduction. Sustainability. 2018;10(6). 10.3390/su10061773.

[78] Cheff I, Nistor I, Palermo D. Pedestrian evacuation modelling of a Canadian West Coast community from a near-field tsunami event. Nat Hazards. 2018;98(1):229–49. 10.1007/s11069-018-3487-5.

[79] Canales JL, Del Río MV, Gubler A. Increasing tsunami risk through intensive urban densification in metropolitan areas: a longitudinal analysis in Viña del Mar, Chile. Int J Disaster Risk Reduct. 2019;41:101312.

[80] Lea CS, Beasley CM, Cox A. Regional after-action review among local health department personnel after Hurricane matthew in eastern North Carolina, United States. J Public Health Manag Pract. 2019 Nov/Dec;25(6):606–09. 10.1097/PHH.0000000000000989. 30969271 10.1097/PHH.0000000000000989

[81] Mostafizi A, Wang H, Cox D, Dong S. An agent-based vertical evacuation model for a near-field tsunami: choice behavior, logical shelter locations, and life safety. Int J Disaster Risk Reduct. 2019;34:467–79. 10.1016/j.ijdrr.2018.12.018.

[82] Valois P, Caron M, Gousse-Lessard AS, Talbot D, Renaud JS. Development and validation of five behavioral indices of flood adaptation. BMC Public Health. 2019 Feb 28;19(1):245. 10.1186/s12889-019-6564-0. 30819122 10.1186/s12889-019-6564-0 PMC6394037

[83] Wilson MJ, Sugg MM, Lane SJ. Identifying multivariate vulnerability of nursing home facilities throughout the southeastern United States. Int J Disaster Risk Reduct. 2019;36. 10.1016/j.ijdrr.2019.101106.

[84] Hofflinger A, Somos-Valenzuela MA, Vallejos-Romero A. Response time to flood events using a social vulnerability index (ReTSVI). Nat Hazards Earth System Sci. 2019;19(1):251–67. 10.5194/nhess-19-251-2019.

[85] Kirby RH, Reams MA, Lam NSN, Zou L, Dekker GGJ, Fundter DQP. Assessing social vulnerability to flood Hazards in the Dutch province of Zeeland. Int J Disaster Risk Sci. 2019;10(2):233–43. 10.1007/s13753-019-0222-0.

[86] Burger J, Gochfeld M. Involving community members in preparedness and resiliency involves bi-directional and iterative communication and actions: a case study of vulnerable populations in New Jersey following superstorm Sandy. J Risk Res. 2020;23(4):541–56. 10.1080/13669877.2019.1593221.

[87] Creach A, Bastidas-Arteaga E, Pardo S, Mercier D. Vulnerability and costs of adaptation strategies for housing subjected to flood risks: application to La Guérinière France. Mar Policy. 2020;117. 10.1016/j.marpol.2019.02.010.

[88] Ersing RL, Pearce C, Collins J, Saunders ME, Polen A. Geophysical and social influences on evacuation decision-making: the case of Hurricane Irma. Atmosphere. 2020;11(8). 10.3390/atmos11080851.

[89] Fan J, Huang G. Evaluation of flood risk management in Japan through a recent case. Sustainability. 2020;12(13). 10.3390/su12135357.

[90] Johnson S, Yu D. From flooding to finance: NHS ambulance-assisted evacuations of care home residents in Norfolk and Suffolk, UK. J Flood Risk Manag. 2020;13(1). 10.1111/jfr3.12592.

[91] Martínez C, Cienfuegos R, Inzunza S, Urrutia A, Guerrero N. Worst-case tsunami scenario in Cartagena Bay, central Chile: challenges for coastal risk management. Ocean Coastal Manag. 2020;185. 10.1016/j.ocecoaman.2019.105060.

[92] Rock M, Blue G. Healthy publics as multi-species matters: solidarity with people’s pets in one Health promotion. Humanit Soc Sci Commun. 2020;7(1). 10.1057/s41599-020-0509-1.

[93] Sritart H, Miyazaki H, Kanbara S, Hara T. Methodology and application of Spatial vulnerability assessment for evacuation shelters in disaster planning. Sustainability. 2020;12(18). 10.3390/su12187355.

[94] Sugiyama T, Yamori K. Consideration of evacuation drills utilizing the capabilities of people with special needs. J Disaster Res. 2020;15(6):794–801. 10.20965/jdr.2020.p0794.

[95] Tammar A, Abosuliman SS, Rahaman KR. Social capital and disaster resilience nexus: a study of flash flood recovery in Jeddah City. Sustainability. 2020;12(11). 10.3390/su12114668.

[96] Tellman B, Schank C, Schwarz B, Howe PD, de Sherbinin A. Using disaster outcomes to validate components of social vulnerability to floods: flood deaths and property damage across the USA. Sustainability. 2020;12(15). 10.3390/su12156006.

[97] Yu D, et al. Disruption of emergency response to vulnerable populations during floods. Nat Sustainability. 2020;3(9):728–36. 10.1038/s41893-020-0516-7.

[98] Lee Y-H, Keum H-J, Han K-Y, Hong W-H. A hierarchical flood shelter location model for walking evacuation planning. Environ Hazards. 2020;20(4):432–55. 10.1080/17477891.2020.1840327.

[99] Alexandrescu F, Anghel I-M, Adorjáni J, Ștefănescu L, Pop A, Mihai A. On the path of evictions and invisibilization: poor Roma facing climate vulnerability. Cities. 2021;114:103201. 10.1016/j.cities.2021.103201.

[100] Bae CY, Kobayashi K. Analysis of evacuation time for vulnerable individuals during inundation of lowland areas. J Disaster Res. 2021;16(5):866–73. 10.20965/jdr.2021.p0866.

[101] Collins J, Polen A, McSweeney K, Colón-Burgos D, Jernigan I. Hurricane risk perceptions and evacuation decision-making in the age of COVID-19. Bull Am Meteorolog Soc. 2021;102(4):E836–48. 10.1175/bams-d-20-0229.1.

[102] Crowley J. Social vulnerability factors and reported post-disaster needs in the aftermath of Hurricane Florence. Int J Disaster Risk Sci. 2020;12(1):13–23. 10.1007/s13753-020-00315-5.

[103] Dargin JS, Li Q, Jawer G, Xiao X, Mostafavi A. Compound hazards: an examination of how hurricane protective actions could increase transmission risk of COVID-19. Int J Disaster Risk Reduct. 2021 Nov;65:102560. 10.1016/j.ijdrr.2021.102560. 34545320 10.1016/j.ijdrr.2021.102560 PMC8443318

[104] Ghosh AK, Mecklenburg M, Ibrahim S, Daniel P. Health care needs in the aftermath of Hurricane Maria in Puerto Rico: a perspective from Federal medical shelter Manati. Prehosp Disaster Med. 2021 Jun;36(3):260–64. 10.1017/S1049023X21000339. 33853696 10.1017/S1049023X21000339

[105] Hill S, et al. Changing logistics of evacuation transportation in hazardous settings during COVID-19. Nat Hazards Rev. 2021;22(3). 10.1061/(asce)nh.1527-6996.0000506.

[106] Hsiao C-C, Sun M-C, Chen AY, Hsu Y-T. Location problems for shelter-in-place deployment: a case study of vertical evacuation upon dam-break floods. Int J Disaster Risk Reduct. 2021;57. 10.1016/j.ijdrr.2021.102048.

[107] Khalafzai M-AK, McGee TK, Parlee B. Spring flooding and recurring evacuations of Kashechewan first Nation, northern Ontario, Canada. Int J Disaster Risk Reduct. 2021;63. 10.1016/j.ijdrr.2021.102443.

[108] Nakai H, Itatani T, Kaganoi S, Okamura A, Horiike R, Yamasaki M. Needs of children with neurodevelopmental disorders and geographic location of emergency shelters suitable for vulnerable people during a tsunami. Int J Environ Res Public Health. 2021;18(4). 10.3390/ijerph18041845. 10.3390/ijerph18041845 PMC7917631 33672833

[109] Nakai H, Itatani T, Horiike R. Construction of an evacuee placement model for tsunami shelters considering physical distancing to prevent COVID-19 infection. Prog Disaster Sci. 2021 Oct;11:100183. 10.1016/j.pdisas.2021.100183. 10.1016/j.pdisas.2021.100183 PMC8205553 34151247

[110] Ohtsu N, Hokugo A, Cruz AM, Sato Y, Araki Y, Park H. Evacuation of vulnerable people during a Natech: a case study of a flood and factory explosion in Japan. Int J Disaster Resilience Built Environ. 2021;14(1):53–67. 10.1108/ijdrbe-04-2021-0043.

[111] Bailie J, Matthews V, Bailie R, Villeneuve M, Longman J. Exposure to risk and experiences of river flooding for people with disability and carers in rural Australia: a cross-sectional survey. BMJ Open. 2022 Aug 2;12(8):e056210. 10.1136/bmjopen-2021-056210. 10.1136/bmjopen-2021-056210 PMC9252212 35918120

[112] Calloway EE, Nugent NB, Stern KL, Mueller A, Yaroch AL. Lessons learned from the 2019 Nebraska floods: implications for emergency management, Mass care, and food Security. Int J Environ Res Public Health. 2022 Sep 9;19(18). 10.3390/ijerph191811345. 10.3390/ijerph191811345 PMC9517450 36141617

[113] Chelariu O-E, Iațu C, Minea I. A GIS-based model for flood shelter locations and Pedestrian evacuation scenarios in a rural mountain catchment in Romania. Water. 2022;14(19). 10.3390/w14193074.

[114] Fraser T. The road more traveled: evacuation networks in the US and Japan. Environ Behav. 2022;54(4):833–63. 10.1177/00139165221090159.

[115] Fraser T. Fleeing the unsustainable city: soft policy and the dual effect of social capital in hurricane evacuation. Sustainability Sci. 2022;17(5):1995–2011. 10.1007/s11625-022-01098-y.

[116] Munawar HS, Mojtahedi M, Hammad AWA, Ostwald MJ, Waller ST. An AI/ML-based strategy for disaster response and evacuation of victims in aged care facilities in the Hawkesbury-Nepean Valley: a perspective. Buildings. 2022;12(1). 10.3390/buildings12010080.

[117] Sakib N, et al. A GIS enhanced data analytics approach for predicting nursing home Hurricane evacuation response. Health Inf Sci Syst. 2022 Dec;10(1):28. 10.1007/s13755-022-00190-y. 10.1007/s13755-022-00190-y PMC9474783 36120113

[118] Sekiguchi H, Takeuchi R, Sato Y, Matsumoto T, Kobayashi J, Umemura T. Can homecare chronic respiratory Disease patients with home oxygen treatment (hot) in Southern Okinawa, Japan Be evacuated ahead of the next anticipated tsunami? Int J Environ Res Public Health. 2022 May 6;19(9). 10.3390/ijerph19095647. 10.3390/ijerph19095647 PMC9103670 35565042

[119] Yang B, Kim M, Lee C, Hwang S, Choi J. Developing an Automated analytical process for disaster response and recovery in communities prone to isolation. Int J Environ Res Public Health. 2022 Oct 27;19(21). 10.3390/ijerph192113995. 10.3390/ijerph192113995 PMC9658131 36360884

[120] Noret C, Sumi T, Mitsunari M, Hamaguchi T, Peyras L. Challenges in flood control operation and dissemination of information - lessons from the record-breaking heavy rain in July 2018, Japan. E3S Web Conf. 2022;346. 10.1051/e3sconf/202234602005.

[121] Fan J, Huang G. Are women more vulnerable to flooding than men in an aging Japanese Society? Int J Environ Res Public Health. 2023 Jan 11;20(2). 10.3390/ijerph20021299. 10.3390/ijerph20021299 PMC9858849 36674055

[122] Gangwal U, Siders AR, Horney J, Michael HA, Dong S. Critical facility accessibility and road criticality assessment considering flood-induced partial failure. Sustain Resilient Infrastruct. 2022;8(sup1):337–55. 10.1080/23789689.2022.2149184.

[123] Karpouza M, Bathrellos GD, Kaviris G, Antonarakou A, Skilodimou HD. How could students be safe during flood and tsunami events? Int J Disaster Risk Reduct. 2023;95. 10.1016/j.ijdrr.2023.103830.

[124] La Greca AM, Burdette ET, Brodar KE. Climate change and extreme weather disasters: evacuation stress is associated with youths’ somatic complaints. Front Psychol. 2023;14:1196419. 10.3389/fpsyg.2023.1196419. 10.3389/fpsyg.2023.1196419 PMC10323363 37425189

[125] Mazumder RK, Enderami SA, Rosenheim N, Sutley EJ, Stanley M, Meyer M. Estimating long-term K-12 student homelessness after a catastrophic flood disaster. Resilient Cities Struct. 2023;2(2):82–92. 10.1016/j.rcns.2023.07.005.

[126] Rickless DS, Wilt GE, Sharpe JD, Molinari N, Stephens W, LeBlanc TT. Social vulnerability and access of local medical care during Hurricane Harvey: a Spatial analysis. Disaster Med Public Health Prep. 2021;17:e12. 10.1017/dmp.2020.421. 10.1017/dmp.2020.421 PMC8440658 33720000

[127] Seeteram NA, Ash K, Sanders BF, Schubert JE, Mach KJ. Modes of climate mobility under sea-level rise. Environ Res Lett. 2023;18(11). 10.1088/1748-9326/acfe22.

[128] Sohn W, Kotval-Karamchandani Z. Risk perception of compound emergencies: a household survey on flood evacuation and sheltering behavior during the COVID-19 pandemic. Sustain Cities Soc. 2023 Jul;94:104553. 10.1016/j.scs.2023.104553. 10.1016/j.scs.2023.104553 PMC10035798 36992858

[129] Tsao T-C, Chen C-Y. Transdisciplinary approach toward preparedness in a mountainous community in central Taiwan and its impact on disaster evacuation: a case study. J Disaster Res. 2023;18(5):456–61. 10.20965/jdr.2023.p0456.

[130] Yang J, et al. Integrating storm surge modeling and accessibility analysis for planning of special-needs hurricane shelters in Panama City, Florida. Transp Plann Technol. 2023;46(2):241–61. 10.1080/03081060.2022.2162053.

[131] Fekete A, Rufat S. Should everyone in need be treated equally? A European survey of expert judgment on social vulnerability to floods and pandemics to validate multi-hazard vulnerability factors. Int J Disaster Risk Reduct. 2023;85:103527. 10.1016/j.ijdrr.2023.103527. 10.1016/j.ijdrr.2023.103527 PMC9817349 36628156

[132] Tedesco M, Foster S, Baptista A, Zuzak C. A multi-hazard climate, displacement and socio-vulnerability score for New York City. Sustainability. 2023;16(1). 10.3390/su16010042.

[133] Anand G, Marcotullio PJ. Spatial disparities in flood vulnerability in New York City. J Hosp Manag Health Policy. 2024;8:4–4. 10.21037/jhmhp-23-92.

[134] Barrionuevo JFS, Noblejas HC, Rodríguez MFM. Mapping tools for flood risk rescue and assistance management. Land. 2024;13(1):68.

[135] Ermagun A, Smith V, Janatabadi F. High urban flood risk and no shelter access disproportionally impacts vulnerable communities in the USA. Commun Earth Environ. 2024;5(1). 10.1038/s43247-023-01165-x.

[136] Bidadian B, Strager MP, Ghadimi H, Sharma M. Flood exposure, vulnerability, and risk distribution in urban areas: application of geospatial data analytics and Index development. GeoHazards. 2024;5(3):833–52. 10.3390/geohazards5030042.

[137] Bogdan E, Krueger R, Wright J, Woods K, Cottar S. Disaster awareness and preparedness among older adults in Canada regarding floods, wildfires, and earthquakes. Int J Disaster Risk Sci. 2024;15(2):198–212. 10.1007/s13753-024-00555-9.

[138] Brennan M, Srini T, Steil J. High and dry: rental markets after flooding disasters. Urban Aff Rev. 2024;60(6):1806–38. 10.1177/10780874241243355.

[139] Han Y, Ye X, Zhu C. The unequal impact of disasters: assessing the interplay between social vulnerability, public assistance, flood insurance, and migration in the U.S. Urban Inf. 2024;3(1):30. 10.1007/s44212-024-00061-9. 10.1007/s44212-024-00061-9 PMC11527954 39493421

[140] Miyatake H, et al. Analysis of the timing of evacuation and associated factors among home health care patients during flooding: a single-clinic-based mixed methods study. Int J Disaster Risk Reduct. 2024;112. 10.1016/j.ijdrr.2024.104762.

[141] Rufat S, Comby E, Lhomme S, Santoni V. Context matters when evacuating large cities: shifting the focus from individual characteristics to location and social vulnerability. Environ Sciamp Policy. 2024;162. 10.1016/j.envsci.2024.103925.

[142] Han Y, Zhai W, Mozumder P, van Westen C, Chen C. Modeling evacuation activities amid compound hazards: insights from hurricane Irma in Southeast Florida. Travel Behaviour Soc. 2025;38. 10.1016/j.tbs.2024.100933.

[143] Li Y, Wang Y, Gong J. An integrated metric for rapid and equitable emergency rescue during urban flash flooding events. Int J Disaster Risk Reduct. 2025;118. 10.1016/j.ijdrr.2025.105209.

[144] Rhein B, Kreibich H. Causes of the exceptionally high number of fatalities in the ahr valley, Germany, during the 2021 flood. Nat Hazards Earth System Sci. 2025;25(2):581–89. 10.5194/nhess-25-581-2025.

[145] Page MJ, et al. The PRISMA, 2020 statement: an updated guideline for reporting systematic reviews. BMJ. 2021;372:n71. 10.1136/bmj.n71. 10.1136/bmj.n71 PMC8005924 33782057

[146] Zarowsky C, Haddad S, Nguyen VK. Beyond ‘vulnerable groups’: contexts and dynamics of vulnerability. Glob Health Promot. 2013 Mar;20(1):3–9, 80–7, 92–9. 10.1177/1757975912470062. 10.1177/1757975912470062 23549696

[147] Napier AD, Volkmann AN, D A, Volkmann A. The vulnerability Vortex: health, exclusion, and social responsibility. In: Anthropology and responsibility. Routledge; 2023. p. 90–109.

[148] Phattharapornjaroen P, Glantz V, Carlström E, Holmqvist LD, Sittichanbuncha Y, Khorram-Manesh A. The feasibility of implementing the flexible surge capacity concept in Bangkok: willing participants and educational gaps. Int J Environ Res Public Health. 2021;18(15):7793. 10.3390/ijerph18157793 PMC8345441 34360083

[149] Khorram-Manesh R, Khorram-Manesh A. Can hotels be used as alternative care sites in disasters and public health emergencies-a narrative review. AIMS Public Health. 2024;11(3):918–36. 10.3934/publichealth.2024047. 10.3934/publichealth.2024047 PMC11474331 39416902

[150] Uscher-Pines L. Health effects of relocation following disaster: a systematic review of the literature. Disasters. 2009 Mar;33(1):1–22. 10.1111/j.1467-7717.2008.01059.x. 18498372 10.1111/j.1467-7717.2008.01059.x

[151] Adams GG, MacMillan L, Smith T, Sharp A, Casagrande R. Meta-analysis on the health effects resulting from evacuation or relocation. Disaster Med Public Health Prep. 2023 Nov 23;17:e538. 10.1017/dmp.2023.55. 37994037 10.1017/dmp.2023.55

[152] Center for Disaster Philanthropy. 2024 Europe floods. https://disasterphilanthropy.org/disasters/2024-central-and-eastern-europe-floods/#:%7E:text=Several%20European%20countries%20flooded%20in,and%20continuing%20for%20several%20days. (accessed 2025).

[153] Durbin A. Italy next to face storm after 21 killed in Europe floods, ed. BBC. 2024.

[154] Jones S. Almost half of Valencia’s flood victims were aged over 70, figures show, ed. The guardian. 2024.

[155] Messaggero I. Devastating floods in Valencia: rising death toll and Ongoing rescue efforts, ed. Il Messaggero. 2024.

