# Supplementary material for: Understanding vulnerability to flood-induced disasters: a comprehensive scoping review on at-risk individuals and evacuation challenges
Source: BMC Health Serv Res. 2025 Dec 18;26:171. doi: 10.1186/s12913-025-13898-w (PMC12870217; doi:10.1186/s12913-025-13898-w)
Supplement: Supplementary file 1 — Supplementary Material 1 [file 12913_2025_13898_MOESM1_ESM.docx]

**Supplementary material I** Search Strategy

**I.a** Search strategy adopted in Scopus

| **Item** | **Search strings** |
| --- | --- |
| #1 | ( TITLE-ABS-KEY ( flood* ) OR TITLE-ABS-KEY ( inundation* ) OR TITLE-ABS-KEY ( landslide* ) OR TITLE-ABS-KEY ( dams ) OR TITLE-ABS-KEY ( embankment ) OR TITLE-ABS-KEY ( overflow* ) OR TITLE-ABS-KEY ( storm surge* ) OR TITLE-ABS-KEY ( storm water* ) |
| #2 | TITLE-ABS-KEY ( evacuat* ) OR TITLE-ABS-KEY ( displace* ) OR TITLE-ABS-KEY ( evacuee* ) OR TITLE-ABS-KEY ( shelter* ) OR TITLE-ABS-KEY ( reception AND cent* ) OR TITLE-ABS-KEY ( alternative AND site* ) OR TITLE-ABS-KEY ( alternative AND care AND site* ) OR TITLE-ABS-KEY ( alternate AND care AND site* ) OR TITLE-ABS-KEY ( alternative AND facilit* ) OR TITLE-ABS-KEY ( alternative AND area* ) |
| #3 | TITLE-ABS-KEY ( vulnerab* ) OR TITLE-ABS-KEY ( fragil* ) OR TITLE-ABS-KEY ( special need* ) ) |
| #4 | #1 AND #2 AND #3 |
| #5 | Filters: from 2014/1/1 - 2024/4/30 |
| #6 | #4 AND #5 |

**I.b** Search strategy adopted in Web of Science

| **Item** | **Search strings** |
| --- | --- |
| #1 | (TS=(flood*) OR TS=(inundation*) OR TS=(landslide*) OR TS=(dams) OR TS=(embankment) OR TS=(overflow*) OR TS=(storm surge*) OR TS=(storm water*)) |
| #2 | (TS=(evacuat*) OR TS=(displace*) OR TS=(evacuee*) OR TS=(shelter*) OR TS=(reception cent*) OR TS=(alternative site*) OR TS=(alternative care site*) OR TS=(alternate care site*) OR TS=(alternative facilit*) OR TS=(alternative area*)) |
| #3 | (TS=(vulnerab*) OR TS=(fragil*) OR TS=(special need*)) |
| #4 | #1 AND #2 AND #3 |
| #5 | Filters: from 2014/1/1 - 2024/4/30 |
| #6 | #4 AND #5 |

**I.c** Search strategy adopted in PubMed

| **Item** | **Search strings** |
| --- | --- |
| #1 | (flood*[Title/Abstract] OR inundation*[Title/Abstract] OR landslide*[Title/Abstract] OR dams*[Title/Abstract] OR embankment[Title/Abstract] OR overflow*[Title/Abstract] OR storm surge*[Title/Abstract] OR storm water*[Title/Abstract]) |
| #2 | (evacuat*[Title/Abstract] OR displace*[Title/Abstract] OR evacuee*[Title/Abstract] OR shelter*[Title/Abstract] OR reception cent*[Title/Abstract] OR alternative site*[Title/Abstract] OR alternative care site*[Title/Abstract] OR alternate care site*[Title/Abstract] OR alternative facilit*[Title/Abstract] OR alternative area*[Title/Abstract]) |
| #3 | (vulnerab*[Title/Abstract] OR fragil*[Title/Abstract] OR special need*[Title/Abstract]) |
| #4 | #1 AND #2 AND #3 |
| #5 | Filters: from 2014/1/1 - 2024/4/30 |
| #6 | #4 AND #5 |

**Supplementary material II** List of economies, World Bank Classifications - Income classifications set on 1 July 2022 remain in effect until 1 July 2023 (in grey countries included in the review)

| **Country/Economy** | **Code** | **Region** | **Income group** | **Classification** |
| --- | --- | --- | --- | --- |
| Afghanistan | AFG | South Asia | Low income | **LIC** |
| Albania | ALB | Europe & Central Asia | Upper middle income | **UMIC** |
| Algeria | DZA | Middle East & North Africa | Lower middle income | **LMIC** |
| American Samoa | ASM | East Asia & Pacific | Upper middle income | **UMIC** |
| Andorra | AND | Europe & Central Asia | High income | **HIC** |
| Angola | AGO | Sub-Saharan Africa | Lower middle income | **LMIC** |
| Antigua and Barbuda | ATG | Latin America & Caribbean | High income | **HIC** |
| Argentina | ARG | Latin America & Caribbean | Upper middle income | **UMIC** |
| Armenia | ARM | Europe & Central Asia | Upper middle income | **UMIC** |
| Aruba | ABW | Latin America & Caribbean | High income | **HIC** |
| Australia | AUS | East Asia & Pacific | High income | **HIC** |
| Austria | AUT | Europe & Central Asia | High income | **HIC** |
| Azerbaijan | AZE | Europe & Central Asia | Upper middle income | **UMIC** |
| Bahamas, The | BHS | Latin America & Caribbean | High income | **HIC** |
| Bahrain | BHR | Middle East & North Africa | High income | **HIC** |
| Bangladesh | BGD | South Asia | Lower middle income | **LMIC** |
| Barbados | BRB | Latin America & Caribbean | High income | **HIC** |
| Belarus | BLR | Europe & Central Asia | Upper middle income | **UMIC** |
| Belgium | BEL | Europe & Central Asia | High income | **HIC** |
| Belize | BLZ | Latin America & Caribbean | Upper middle income | **UMIC** |
| Benin | BEN | Sub-Saharan Africa | Lower middle income | **LMIC** |
| Bermuda | BMU | North America | High income | **HIC** |
| Bhutan | BTN | South Asia | Lower middle income | **LMIC** |
| Bolivia | BOL | Latin America & Caribbean | Lower middle income | **LMIC** |
| Bosnia and Herzegovina | BIH | Europe & Central Asia | Upper middle income | **UMIC** |
| Botswana | BWA | Sub-Saharan Africa | Upper middle income | **UMIC** |
| Brazil | BRA | Latin America & Caribbean | Upper middle income | **UMIC** |
| British Virgin Islands | VGB | Latin America & Caribbean | High income | **HIC** |
| Brunei Darussalam | BRN | East Asia & Pacific | High income | **HIC** |
| Bulgaria | BGR | Europe & Central Asia | Upper middle income | **UMIC** |
| Burkina Faso | BFA | Sub-Saharan Africa | Low income | **LIC** |
| Burundi | BDI | Sub-Saharan Africa | Low income | **LIC** |
| Cabo Verde | CPV | Sub-Saharan Africa | Lower middle income | **LMIC** |
| Cambodia | KHM | East Asia & Pacific | Lower middle income | **LMIC** |
| Cameroon | CMR | Sub-Saharan Africa | Lower middle income | **LMIC** |
| Canada | CAN | North America | High income | **HIC** |
| Cayman Islands | CYM | Latin America & Caribbean | High income | **HIC** |
| Central African Republic | CAF | Sub-Saharan Africa | Low income | **LIC** |
| Chad | TCD | Sub-Saharan Africa | Low income | **LIC** |
| Channel Islands | CHI | Europe & Central Asia | High income | **HIC** |
| Chile | CHL | Latin America & Caribbean | High income | **HIC** |
| China | CHN | East Asia & Pacific | Upper middle income | **UMIC** |
| Colombia | COL | Latin America & Caribbean | Upper middle income | **UMIC** |
| Comoros | COM | Sub-Saharan Africa | Lower middle income | **LMIC** |
| Congo, Dem. Rep. | COD | Sub-Saharan Africa | Low income | **LIC** |
| Congo, Rep. | COG | Sub-Saharan Africa | Lower middle income | **LMIC** |
| Costa Rica | CRI | Latin America & Caribbean | Upper middle income | **UMIC** |
| Côte d’Ivoire | CIV | Sub-Saharan Africa | Lower middle income | **LMIC** |
| Croatia | HRV | Europe & Central Asia | High income | **HIC** |
| Cuba | CUB | Latin America & Caribbean | Upper middle income | **UMIC** |
| Curaçao | CUW | Latin America & Caribbean | High income | **HIC** |
| Cyprus | CYP | Europe & Central Asia | High income | **HIC** |
| Czech Republic | CZE | Europe & Central Asia | High income | **HIC** |
| Denmark | DNK | Europe & Central Asia | High income | **HIC** |
| Djibouti | DJI | Middle East & North Africa | Lower middle income | **LMIC** |
| Dominica | DMA | Latin America & Caribbean | Upper middle income | **UMIC** |
| Dominican Republic | DOM | Latin America & Caribbean | Upper middle income | **UMIC** |
| Ecuador | ECU | Latin America & Caribbean | Upper middle income | **UMIC** |
| Egypt, Arab Rep. | EGY | Middle East & North Africa | Lower middle income | **LMIC** |
| El Salvador | SLV | Latin America & Caribbean | Lower middle income | **LMIC** |
| Equatorial Guinea | GNQ | Sub-Saharan Africa | Upper middle income | **UMIC** |
| Eritrea | ERI | Sub-Saharan Africa | Low income | **LIC** |
| Estonia | EST | Europe & Central Asia | High income | **HIC** |
| Eswatini | SWZ | Sub-Saharan Africa | Lower middle income | **LMIC** |
| Ethiopia | ETH | Sub-Saharan Africa | Low income | **LIC** |
| Faroe Islands | FRO | Europe & Central Asia | High income | **HIC** |
| Fiji | FJI | East Asia & Pacific | Upper middle income | **UMIC** |
| Finland | FIN | Europe & Central Asia | High income | **HIC** |
| France | FRA | Europe & Central Asia | High income | **HIC** |
| French Polynesia | PYF | East Asia & Pacific | High income | **HIC** |
| Gabon | GAB | Sub-Saharan Africa | Upper middle income | **UMIC** |
| Gambia, The | GMB | Sub-Saharan Africa | Low income | **LIC** |
| Georgia | GEO | Europe & Central Asia | Upper middle income | **UMIC** |
| Germany | DEU | Europe & Central Asia | High income | **HIC** |
| Ghana | GHA | Sub-Saharan Africa | Lower middle income | **LMIC** |
| Gibraltar | GIB | Europe & Central Asia | High income | **HIC** |
| Greece | GRC | Europe & Central Asia | High income | **HIC** |
| Greenland | GRL | Europe & Central Asia | High income | **HIC** |
| Grenada | GRD | Latin America & Caribbean | Upper middle income | **UMIC** |
| Guam | GUM | East Asia & Pacific | High income | **HIC** |
| Guatemala | GTM | Latin America & Caribbean | Upper middle income | **UMIC** |
| Guinea | GIN | Sub-Saharan Africa | Low income | **LIC** |
| Guinea-Bissau | GNB | Sub-Saharan Africa | Low income | **LIC** |
| Guyana | GUY | Latin America & Caribbean | Upper middle income | **UMIC** |
| Haiti | HTI | Latin America & Caribbean | Lower middle income | **LMIC** |
| Honduras | HND | Latin America & Caribbean | Lower middle income | **LMIC** |
| Hong Kong SAR, China | HKG | East Asia & Pacific | High income | **HIC** |
| Hungary | HUN | Europe & Central Asia | High income | **HIC** |
| Iceland | ISL | Europe & Central Asia | High income | **HIC** |
| India | IND | South Asia | Lower middle income | **LMIC** |
| Indonesia | IDN | East Asia & Pacific | Lower middle income | **LMIC** |
| Iran, Islamic Rep. | IRN | Middle East & North Africa | Lower middle income | **LMIC** |
| Iraq | IRQ | Middle East & North Africa | Upper middle income | **UMIC** |
| Ireland | IRL | Europe & Central Asia | High income | **HIC** |
| Isle of Man | IMN | Europe & Central Asia | High income | **HIC** |
| Israel | ISR | Middle East & North Africa | High income | **HIC** |
| Italy | ITA | Europe & Central Asia | High income | **HIC** |
| Jamaica | JAM | Latin America & Caribbean | Upper middle income | **UMIC** |
| Japan | JPN | East Asia & Pacific | High income | **HIC** |
| Jordan | JOR | Middle East & North Africa | Upper middle income | **UMIC** |
| Kazakhstan | KAZ | Europe & Central Asia | Upper middle income | **UMIC** |
| Kenya | KEN | Sub-Saharan Africa | Lower middle income | **LMIC** |
| Kiribati | KIR | East Asia & Pacific | Lower middle income | **LMIC** |
| Korea, Dem. People's Rep. | PRK | East Asia & Pacific | Low income | **LIC** |
| Korea, Rep. | KOR | East Asia & Pacific | High income | **HIC** |
| Kosovo | XKX | Europe & Central Asia | Upper middle income | **UMIC** |
| Kuwait | KWT | Middle East & North Africa | High income | **HIC** |
| Kyrgyz Republic | KGZ | Europe & Central Asia | Lower middle income | **LMIC** |
| Lao PDR | LAO | East Asia & Pacific | Lower middle income | **LMIC** |
| Latvia | LVA | Europe & Central Asia | High income | **HIC** |
| Lebanon | LBN | Middle East & North Africa | Lower middle income | **LMIC** |
| Lesotho | LSO | Sub-Saharan Africa | Lower middle income | **LMIC** |
| Liberia | LBR | Sub-Saharan Africa | Low income | **LIC** |
| Libya | LBY | Middle East & North Africa | Upper middle income | **UMIC** |
| Liechtenstein | LIE | Europe & Central Asia | High income | **HIC** |
| Lithuania | LTU | Europe & Central Asia | High income | **HIC** |
| Luxembourg | LUX | Europe & Central Asia | High income | **HIC** |
| Macao SAR, China | MAC | East Asia & Pacific | High income | **HIC** |
| Madagascar | MDG | Sub-Saharan Africa | Low income | **LIC** |
| Malawi | MWI | Sub-Saharan Africa | Low income | **LIC** |
| Malaysia | MYS | East Asia & Pacific | Upper middle income | **UMIC** |
| Maldives | MDV | South Asia | Upper middle income | **UMIC** |
| Mali | MLI | Sub-Saharan Africa | Low income | **LIC** |
| Malta | MLT | Middle East & North Africa | High income | **HIC** |
| Marshall Islands | MHL | East Asia & Pacific | Upper middle income | **UMIC** |
| Mauritania | MRT | Sub-Saharan Africa | Lower middle income | **LMIC** |
| Mauritius | MUS | Sub-Saharan Africa | Upper middle income | **UMIC** |
| Mexico | MEX | Latin America & Caribbean | Upper middle income | **UMIC** |
| Micronesia, Fed. Sts. | FSM | East Asia & Pacific | Lower middle income | **LMIC** |
| Moldova | MDA | Europe & Central Asia | Upper middle income | **UMIC** |
| Monaco | MCO | Europe & Central Asia | High income | **HIC** |
| Mongolia | MNG | East Asia & Pacific | Lower middle income | **LMIC** |
| Montenegro | MNE | Europe & Central Asia | Upper middle income | **UMIC** |
| Morocco | MAR | Middle East & North Africa | Lower middle income | **LMIC** |
| Mozambique | MOZ | Sub-Saharan Africa | Low income | **LIC** |
| Myanmar | MMR | East Asia & Pacific | Lower middle income | **LMIC** |
| Namibia | NAM | Sub-Saharan Africa | Upper middle income | **UMIC** |
| Nauru | NRU | East Asia & Pacific | High income | **HIC** |
| Nepal | NPL | South Asia | Lower middle income | **LMIC** |
| Netherlands | NLD | Europe & Central Asia | High income | **HIC** |
| New Caledonia | NCL | East Asia & Pacific | High income | **HIC** |
| New Zealand | NZL | East Asia & Pacific | High income | **HIC** |
| Nicaragua | NIC | Latin America & Caribbean | Lower middle income | **LMIC** |
| Niger | NER | Sub-Saharan Africa | Low income | **LIC** |
| Nigeria | NGA | Sub-Saharan Africa | Lower middle income | **LMIC** |
| North Macedonia | MKD | Europe & Central Asia | Upper middle income | **UMIC** |
| Northern Mariana Islands | MNP | East Asia & Pacific | High income | **HIC** |
| Norway | NOR | Europe & Central Asia | High income | **HIC** |
| Oman | OMN | Middle East & North Africa | High income | **HIC** |
| Pakistan | PAK | South Asia | Lower middle income | **LMIC** |
| Palau | PLW | East Asia & Pacific | Upper middle income | **UMIC** |
| Panama | PAN | Latin America & Caribbean | High income | **HIC** |
| Papua New Guinea | PNG | East Asia & Pacific | Lower middle income | **LMIC** |
| Paraguay | PRY | Latin America & Caribbean | Upper middle income | **UMIC** |
| Peru | PER | Latin America & Caribbean | Upper middle income | **UMIC** |
| Philippines | PHL | East Asia & Pacific | Lower middle income | **LMIC** |
| Poland | POL | Europe & Central Asia | High income | **HIC** |
| Portugal | PRT | Europe & Central Asia | High income | **HIC** |
| Puerto Rico | PRI | Latin America & Caribbean | High income | **HIC** |
| Qatar | QAT | Middle East & North Africa | High income | **HIC** |
| Romania | ROU | Europe & Central Asia | High income | **HIC** |
| Russian Federation | RUS | Europe & Central Asia | Upper middle income | **UMIC** |
| Rwanda | RWA | Sub-Saharan Africa | Low income | **LIC** |
| Samoa | WSM | East Asia & Pacific | Lower middle income | **LMIC** |
| San Marino | SMR | Europe & Central Asia | High income | **HIC** |
| São Tomé and Príncipe | STP | Sub-Saharan Africa | Lower middle income | **LMIC** |
| Saudi Arabia | SAU | Middle East & North Africa | High income | **HIC** |
| Senegal | SEN | Sub-Saharan Africa | Lower middle income | **LMIC** |
| Serbia | SRB | Europe & Central Asia | Upper middle income | **UMIC** |
| Seychelles | SYC | Sub-Saharan Africa | High income | **HIC** |
| Sierra Leone | SLE | Sub-Saharan Africa | Low income | **LIC** |
| Singapore | SGP | East Asia & Pacific | High income | **HIC** |
| Sint Maarten (Dutch part) | SXM | Latin America & Caribbean | High income | **HIC** |
| Slovak Republic | SVK | Europe & Central Asia | High income | **HIC** |
| Slovenia | SVN | Europe & Central Asia | High income | **HIC** |
| Solomon Islands | SLB | East Asia & Pacific | Lower middle income | **LMIC** |
| Somalia | SOM | Sub-Saharan Africa | Low income | **LIC** |
| South Africa | ZAF | Sub-Saharan Africa | Upper middle income | **UMIC** |
| South Sudan | SSD | Sub-Saharan Africa | Low income | **LIC** |
| Spain | ESP | Europe & Central Asia | High income | **HIC** |
| Sri Lanka | LKA | South Asia | Lower middle income | **LMIC** |
| St. Kitts and Nevis | KNA | Latin America & Caribbean | High income | **HIC** |
| St. Lucia | LCA | Latin America & Caribbean | Upper middle income | **UMIC** |
| St. Martin (French part) | MAF | Latin America & Caribbean | High income | **HIC** |
| St. Vincent and the Grenadines | VCT | Latin America & Caribbean | Upper middle income | **UMIC** |
| Sudan | SDN | Sub-Saharan Africa | Low income | **LIC** |
| Suriname | SUR | Latin America & Caribbean | Upper middle income | **UMIC** |
| Sweden | SWE | Europe & Central Asia | High income | **HIC** |
| Switzerland | CHE | Europe & Central Asia | High income | **HIC** |
| Syrian Arab Republic | SYR | Middle East & North Africa | Low income | **LIC** |
| Taiwan, China | TWN | East Asia & Pacific | High income | **HIC** |
| Tajikistan | TJK | Europe & Central Asia | Lower middle income | **LMIC** |
| Tanzania | TZA | Sub-Saharan Africa | Lower middle income | **LMIC** |
| Thailand | THA | East Asia & Pacific | Upper middle income | **UMIC** |
| Timor-Leste | TLS | East Asia & Pacific | Lower middle income | **LMIC** |
| Togo | TGO | Sub-Saharan Africa | Low income | **LIC** |
| Tonga | TON | East Asia & Pacific | Upper middle income | **UMIC** |
| Trinidad and Tobago | TTO | Latin America & Caribbean | High income | **HIC** |
| Tunisia | TUN | Middle East & North Africa | Lower middle income | **LMIC** |
| Türkiye | TUR | Europe & Central Asia | Upper middle income | **UMIC** |
| Turkmenistan | TKM | Europe & Central Asia | Upper middle income | **UMIC** |
| Turks and Caicos Islands | TCA | Latin America & Caribbean | High income | **HIC** |
| Tuvalu | TUV | East Asia & Pacific | Upper middle income | **UMIC** |
| Uganda | UGA | Sub-Saharan Africa | Low income | **LIC** |
| Ukraine | UKR | Europe & Central Asia | Lower middle income | **LMIC** |
| United Arab Emirates | ARE | Middle East & North Africa | High income | **HIC** |
| United Kingdom | GBR | Europe & Central Asia | High income | **HIC** |
| United States | USA | North America | High income | **HIC** |
| Uruguay | URY | Latin America & Caribbean | High income | **HIC** |
| Uzbekistan | UZB | Europe & Central Asia | Lower middle income | **LMIC** |
| Vanuatu | VUT | East Asia & Pacific | Lower middle income | **LMIC** |
| Venezuela, RB | VEN | Latin America & Caribbean |  | **See Notes** |
| Vietnam | VNM | East Asia & Pacific | Lower middle income | **LMIC** |
| Virgin Islands (U.S.) | VIR | Latin America & Caribbean | High income | **HIC** |
| West Bank and Gaza | PSE | Middle East & North Africa | Lower middle income | **LMIC** |
| Yemen, Rep. | YEM | Middle East & North Africa | Low income | **LIC** |
| Zambia | ZMB | Sub-Saharan Africa | Low income | **LIC** |
| Zimbabwe | ZWE | Sub-Saharan Africa | Lower middle income | **LMIC** |

**Supplementary material III** Data extraction tool

| **Thematic group** | **Search strings** |
| --- | --- |
| **Study characteristics** | First author |
|  | Title |
|  | Year |
|  | DOI |
|  | Country |
|  | Approach |
|  | Method |
| **Type of event** | Flood |
|  | Hurricane |
|  | Typhoon |
|  | Cyclone |
|  | Tsunami |
|  | Other |
| **Evacuation preparedness** | Surge capacity *(staff, stuff, structure, system)* |
|  | Critical services |
|  | Alert, early warning and rescue |
|  | Coordination |
|  | Information and communication |
|  | Mapping (resources, facilities and people) |
|  | Transport, routes |
|  | Preventive evacuations |
| **Evacuation response** | Surge capacity *(staff, stuff, structure, system)* |
|  | Critical services (*impact, access*) |
|  | Alert, early warning and rescue |
|  | Coordination |
|  | Information and communication |
|  | Mapping (resources, facilities and people) |
|  | Displacement |
|  | Transport, routes |
|  | Evacuees’ behaviour |
|  | Health evacuation needs |
|  | Social evacuation needs |
| **Evacuation recovery** | Recovery evacuation actions |
| **Evacuation process recommendations** | Surge capacity *(staff, stuff, structure, system)* |
|  | Critical services (*impact, access*) |
|  | Alert, early warning and rescue |
|  | Coordination |
|  | Information and communication |
|  | Mapping (resources, facilities and people) |
|  | Displacement |
|  | Transport, routes |
|  | Evacuees’ behaviour |
|  | Preventive evacuations |
|  | Health evacuation needs |
|  | Social evacuation needs |
|  | Recovery evacuation actions |
| **Vulnerability and fragility** | Vulnerability definition and dimensions |
|  | Vulnerable groups |
|  | Vulnerable groups’ special needs |
|  | Recommendations |

**Supplementary material IV** Vulnerability indexes mentioned in the included studies

| **Index** | **References** | **Description** |
| --- | --- | --- |
| Social Vulnerability Index (SoVI) | [54, 55, 66, 84, 89, 92, 97, 123, 133, 137] | Developed by Cutter et al. in 2003, emerged as the most frequently cited multidimensional tool for assessing disaster vulnerability in the reviewed studies. Designed to measure social vulnerability to environmental hazards, SoVI utilizes a principal component analysis (PCA) methodology to synthesize over 30 socio-demographic variables into a composite social vulnerability measure. These variables include poverty, race, gender, age, disability, employment, education, income, single-parent households, home value and ownership, health insurance coverage, hospitals per capita, English proficiency, vehicle access, and characteristics of the built environment, among others. SoVI, leveraging census data, enables assessments of vulnerability at the regional and county levels, facilitating the identification of communities most vulnerable to disasters. This index and the quantitative measures resulting from it have underscored the dynamic nature of vulnerability, highlighting how it can vary significantly across areas exposed to the same hazard, but with different composite levels of social vulnerability. SoVI has been widely recognized and utilized for sub-national vulnerability assessments. |
| CDC Social Vulnerability Index (SVI) | [97, 104, 123, 127, 134, 137] | Evaluates disaster-related social vulnerability levels–at the census level, incorporating four key themes: socioeconomic status, household composition, minority status and language, and housing type and transportation. Each theme includes specific variables such as poverty, unemployment, age, minority status, lack of adequate housing, and transportation limitations. While less frequently cited than SoVI, CDC SVI remains an important tool for identifying vulnerable communities requiring extra support and attention in the face of disasters. |
| Social Vulnerability Score (SVS) | [126] | Developed by Enderami and Sutley, is a flexible composite measure specifically designed to evaluate household-level social vulnerability. The SVS integrates a range of demographic variables such as race, ethnicity, poverty, education, age, and disability status, producing a score to measure vulnerability across communities at the census block level. |
| US Social Vulnerability Index | [74] | Focuses on assessing socioeconomic variables that diminish individuals’ capacities to manage emergencies. |
| Climate Displacement and Socio-Vulnerability (CDSV) score | [133] | Developed for New York City to evaluate intersections between extreme weather events, displacement risk and social vulnerability. It is derived from publicly accessible datasets and integrates components such as the Displacement Risk Index from the New York City Department of City Planning, population vulnerability variables (e.g., race, ethnicity, income, rent proportion on income), housing conditions (e.g., quality of housing), and market pressures, such as the challenges faced by lower-income residents in living in safer areas. |
| Hazard Exposure Vulnerability Index (HazVI) | [66] | N/A |
| Built Environment Vulnerability Index (BEVI) | [66] | N/A |
| Hazards Level Index (HLI) | [84] | Relies on several datasets such as the Homeland Infrastructure Foundation Level Database (HIFLD), the National Oceanic and Atmospheric Administration (NOAA), and the Federal Emergency Management Agency (FEMA). The HLI quantifies hazard exposure by assessing the frequency of events at the census or county level. |
| Community Disaster Resilience Index (CDRI) | [97] | N/A |
| Community Level Index (CLI) | [84] | Constructed using data from the US census and, starting from the SoVI model variables, it produced 7 main components through principal component analysis (PCA): poverty and minority population, age, income and housing, Hispanic population, family status, employment and female population, and nursing facility population. |
| Nursing Home Level Index (NHLI) | [84] | Evaluates nursing home residents’ vulnerability. NHLI incorporates variables derived from the Centers for Medicare & Medicaid Services, Nursing Home Compare Minimum Data Set (MDS)–related to the functional, emotional, cognitive, and disease characteristics of residents, information retrieved from public staffing files and takes into account nursing homes ownership types (e.g., for-profit, non-profit), vaccination rates, staffing hours, as proxies for quality of care. |
| Social Flood Vulnerability Index (SFVI) | [91] | Identifies socially vulnerable individuals to flood risk, highlighting populations such as care home residents – where the majority have disabilities, and cognitive impairments and require continuous personal care. |
| Acuindex | [118] | Provides a composite measure to capture the health conditions and needs of nursing home residents. It is useful to measure the proportion of residents requiring special assistance in daily activities, such as eating, toileting, and transferring from bed, but also those requiring specialized treatments, such as respiratory support, suctioning, etc. |
| Flood Vulnerability Index | [118] | N/A |
| Social Flood Vulnerability Index (SFVI) | [91] | Identifies socially vulnerable individuals to flood risk, highlighting populations such as care home residents – where the majority have disabilities, and cognitive impairments and require continuous personal care. |
| Coastal City Flood Vulnerability Index (CCFVI) | [133] | Devised by Balica et al. to evaluate the vulnerability of coastal areas to flooding, by examining exposure, susceptibility and resilience. |
| Extreme Inherent Vulnerability (VIE) Index | [88] | Introduced by Creach to assess the vulnerability of residential buildings exposed to coastal flood risks. VIE evaluates four criteria: potential floodwater levels inside a building, proximity to flood defenses, architectural type (e.g., single or multi-storey), and accessibility to rescue areas. Buildings are scored according to these criteria, with higher scores reflecting higher vulnerability. The results are clustered into four categories: green (no vulnerability), yellow (low risk), red (moderate risk for vulnerable populations–young, elderly, those with disabilities), and black (high likelihood of fatalities). |
| Tsunami Risk Index | [80] | Introduced to assess tsunami risk levels in Población Vergara, Chile–integrates hazard, exposure, susceptibility, and coping capacity dimensions. |
| Social Vulnerability for Evacuation Assistance Index (SVEAI) | [55] | Developed by Chakraborty et al. to assess social vulnerability specifically related to evacuation needs during disasters. |
| Evacuation Vulnerability Index | [66] | Focuses on individual limitations and the adequacy of public transportation systems to assess evacuation vulnerability. Higher scores reflect greater proportions of disadvantaged populations, longer distances to evacuation to pick-up points, or lower elevation levels, which collectively heighten vulnerability levels. |
| Resilience Capacity Index (RCI) | [97] | N/A |
| Response Time by Social Vulnerability Index (ReTSVI) | [85] | Combines evacuation rate curves, mobilization data, inundation models, and social vulnerability indices, and generates detailed maps indicating evacuation rates across various locations, thereby aiding in identifying areas where populations may require additional support during evacuations. |
